# Comparative genomic and transcriptome analyses of two *Pectobacterium brasiliense* strains revealed distinct virulence determinants and phenotypic features

**DOI:** 10.3389/fmicb.2024.1362283

**Published:** 2024-05-10

**Authors:** Yue Sun, Handique Utpal, Yajuan Wu, Qinghua Sun, Zhiwen Feng, Yue Shen, Ruofang Zhang, Xiaofeng Zhou, Jian Wu

**Affiliations:** ^1^Potato Engineering and Technology Research Center, Inner Mongolia University, Hohhot, Inner Mongolia, China; ^2^BGI Research, Shenzhen, China; ^3^State Key Laboratory of Biocontrol, Guangdong Provincial Key Laboratory of Plant Resources, School of Agriculture and Biotechnology, Sun Yat-sen University, Shenzhen, China

**Keywords:** *Pectobacterium brasiliense*, potato soft rot, endoglucanase, bacterial virulence, comparative genomics

## Abstract

Potato soft rot caused by *Pectobacterium* spp. are devastating diseases of potato which cause severe economic losses worldwide. *Pectobacterium brasiliense* is considered as one of the most virulent species. However, the virulence mechanisms and pathogenicity factors of this strain have not been fully elucidated. Here, through pathogenicity screening, we identified two *Pectobacterium brasiliense* isolates, SM and DQ, with distinct pathogenicity levels. SM exhibits higher virulence compared to DQ in inducing aerial stem rot, blackleg and tuber soft rot. Our genomic and transcriptomic analyses revealed that SM encodes strain specific genes with regard to plant cell wall degradation and express higher level of genes associated with bacterial motility and secretion systems. Our plate assays verified higher pectinase, cellulase, and protease activities, as well as fast swimming and swarming motility in SM. Importantly, a unique endoglucanase S specific to SM was identified. Expression of this cellulase in DQ greatly enhances its virulence compared to wild type strain. Our study sheds light on possible determinants causing different pathogenicity of *Pectobacterium brasiliense* species with close evolutionary distance and provides new insight into the direction of genome evolution in response to host variation and environmental stimuli.

## Background

The bacterial blackleg, aerial stem rot and tuber soft rot are devastating potato diseases worldwide (Handique et al., 2022). These diseases are primarily caused by *Pectobacterium* spp. and *Dickeya* spp. These bacterial pathogens secrete plant cell wall-degradation enzymes (PCWDEs) through the type II secretion system (T2SS). These enzymes function to degrade pectin found in the middle lamellae and primary plant cell walls, leading to tissue maceration, often resulting in a moist and foul-smelling rot of plant organs (Hugouvieux-Cotte-Pattat et al., 2014). While all *Pectobacterium* spp. are capable of inducing tuber soft rot (van der Wolf et al., 2017), only specific strains cause blackleg. Notably, *Pectobacterium brasiliense* strains typically exhibit high pathogenicity and can induce blackleg, aerial stem rot, and tuber soft rot. In contrast, *Pectobacterium carotovorum* strains do not induce blackleg (Charkowski, 2018). Originally identified in Brazil (Duarte et al., 2004), *Pectobacterium brasiliense* has emerged as a global concern, with reported cases across various countries in North America, Europe, Africa and East Asia (de Werra et al., 2015; Zhao et al., 2018; Fujimoto, 2022).

Bacterial secretion systems are essential in pathogen-host interaction. The type II secretion system (T2SS) plays a pivotal role in bacterial virulence and the disease cycle, primarily through the secretion of PCWDEs (Mashavha, 2013). These enzymes include pectinases, cellulases, and proteases. The degradation of plant cell wall creates openings that facilitates the entry of bacterial pathogens into the host cell and might also provide carbon sources for bacterial viability. Notably, various virulence factors, such as the necrosis-inducing protein NipE and the AvrL-AvrM proteins, are translocated via T2SS and potentially participate in interactions with plants (Kazemi-Pour et al., 2004). While the type III secretion system (T3SS) is recognized as crucial for virulence in many plant-pathogenic bacteria (Alfano and Collmer, 2004). Studies have shown that T3SS-deficient *Pectobacterium* strains exhibit comparable virulence to wild-type strains in plants (Kim et al., 2009). Moreover, Type IV, V, and VI secretion systems are highly diverse in *Pectobacterium*. The type VI secretion system (T6SS) may also be important in the pathogenicity, which is largely related to various biological functions such as biofilm formation, host adaptation, and bacterial survival (Masum et al., 2017).

Comparative genomic analysis of different *Pectobacterium* spp. has greatly facilitated our understanding of the virulence factors and host-bacteria interactions. The comparative analysis on *Pectobacterium carotovorum* ICMP 5702 and other pathogenic bacteria unveiled orthologous clusters that could be crucial for delineating the evolutionary history of the bacteria and identifying key virulent determinants (Mallick et al., 2022). Genome comparison unveiled a high conservation of most virulence genes among *Pectobacterium* strains, particularly those key determinants involved in the biosynthesis of extracellular enzymes, secretion systems and chemotactic genes (Li et al., 2019). Transcriptome analysis of bacterial pathogens provides a powerful approach to identify and analyze differentially expressed gene patterns during infection. Previous studies successfully identified specific bacterial transcriptomic signatures that are influenced by plant immune activation by establishing two methods for in planta bacterial transcriptome analysis using RNA sequencing (Nobori et al., 2018). Studies have been reported such in plant analysis for various pathogenic bacteria (Chapelle et al., 2015). A study on the role of *D. dadantii* strain 3937 PecS global regulators in early colonization of leaf tissue found more than 600 genes among its regulators (Pédron et al., 2018). Transcriptomics-based approaches can help explore key strategies for interactions between plant pathogens and key physiological processes in plants.

Here we conducted comparative genomic and transcriptomic analyses of SM and DQ with significantly divergent pathogenicity to understand the difference in virulence with a particular focus on virulence factors including plant cell wall-degrading enzymes and secretion systems. We observed that SM with strong pathogenicity harbored a higher number of T2SS machinery and substrate genes, which were upregulated in host plants compared to DQ. Besides, genes encoding flagella assembly were expressed at higher levels in SM compared to DQ under conditions of both plant and nutrient broth. Plate assays confirmed that SM exhibited greater motility compared to DQ, and that SM had higher activity of pectinase, protease and cellulase. Furthermore, SM possessed a unique endoglucanase S gene that was specifically upregulated in host. We experimentally verified the role of endoglucanase S in bacterial pathogenicity by expressing this gene in DQ strain to enhance its virulence. Characterization of these functional determinants and clarifying the potential virulence factors of *Pectobacterium brasiliense* will help to develop comprehensive prevention and control measures for potato bacterial soft rot.

## Results

### *Pectobacterium brasiliense* SM is more virulent than DQ strain on multiple potato varieties

**Figure 1:**
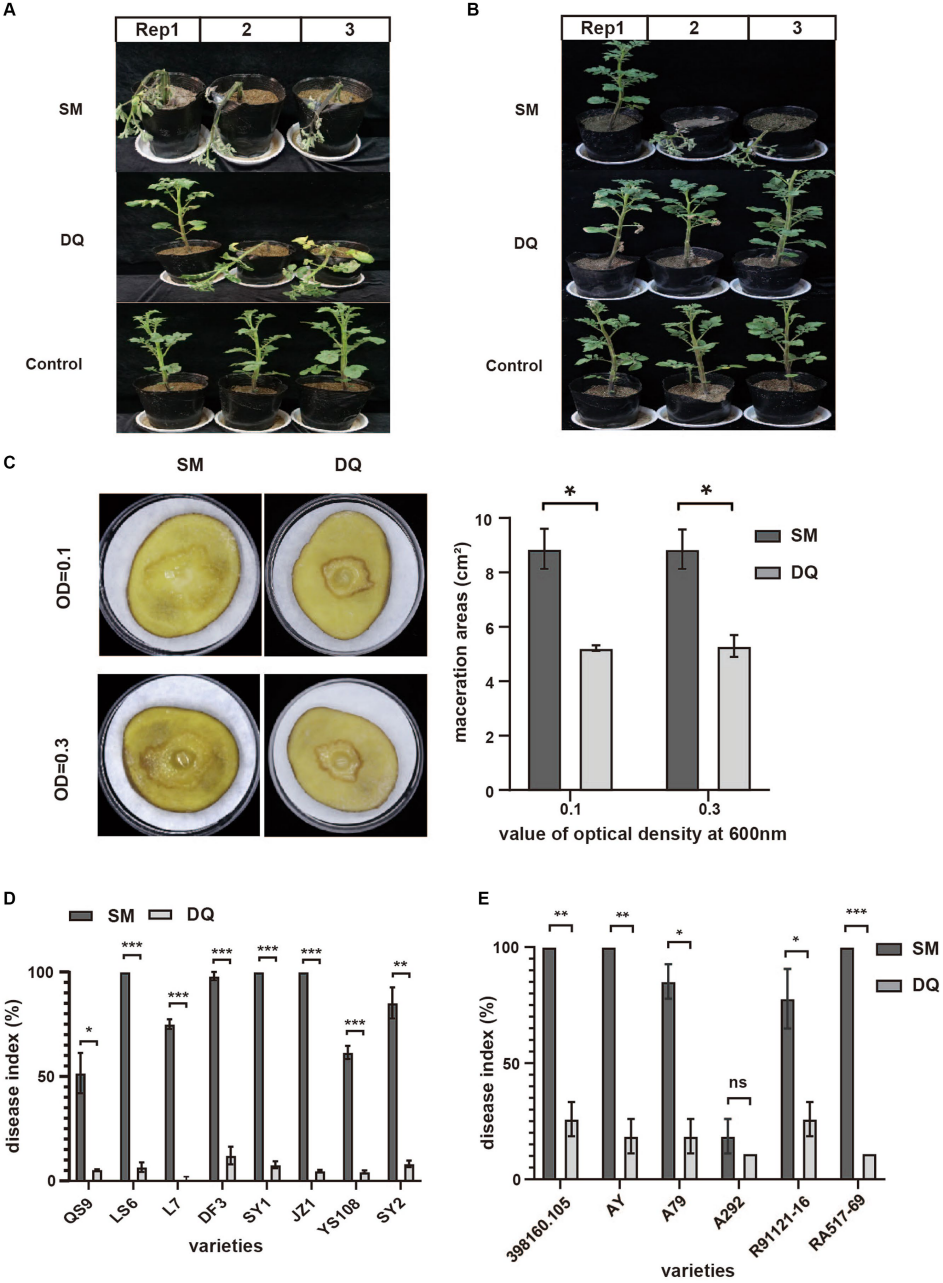
The pathogenicity of *Pectobacterium brasiliense* SM and DQ strains. **(A)** Pathogenicity assay of SM and DQ causing aerial stem rot on potatoes. Bacterial suspensions (10^8^ CFU/mL) were injected into potato stem by a syringe. Photographs were taken 3 days after inoculation. **(B)** Pathogenicity assay of SM and DQ causing blackleg on potatoes. Bacterial suspensions (10^8^ CFU/mL) were inoculated to each pot by soil drench approach. Photographs were taken 2 weeks after infection. **(C)** Potato soft rot assay caused by SM and DQ of OD = 0.1 and 0.3. Maceration areas of potato slice were measured after 24 h infection. **(D)** Disease index of SM and DQ inoculated domestic cultivars, including QS9, LS6, L7, DF3, SY1, JZ1, YS108, and SY2. **(E)** Disease index of SM and DQ inoculated foreign resource varieties, including 398160.105, AY, A79, A292, R91121-16 and RA517-69. 398160.105 is the variety from Peru. AY is an introduced variety from Australia. A79 and A292 are a series of resource species from Argentina. R91121-16 and RA517-69 are introduced varieties from Chile. Means ± SE errors are indicated, *n* ≥ 3 for **(C–E)**. The asterisk represents a significant difference between SM and DQ from Student’s t-test (*p* < 0.05).

In previous study, we identified two *Pectobacterium brasiliense* isolates, SM and DQ, with distinct pathogenicity levels. To comprehensively characterize the phenotypic traits of these strains, their virulence was evaluated using three distinct infection modes. When the stem base is injected, SM inoculated plants exhibited rapid withering and desiccating, indicating higher virulence compared to DQ in inducing aerial stem rot (Figure 1A). Using soil drenching approach, we found that SM had higher pathogenicity compared to DQ in inducing potato blackleg (Figure 1B). Plants inoculated with SM strain showed stem base darkening and wilting of the leaves within 2 weeks. The maceration areas observed on potato slices inoculated with SM are consistently larger compared to those inoculated with DQ (Figure 1C).

To further confirm that this virulence difference is not specific to one potato cultivar., we conducted pathogenicity assays using stem injection on multiple cultivated varieties and lab-preserved foreign resources (Figures 1D,E). Regarding the main domestic varieties, QS9 (Qingshu No. 9), YS108 (Yunshu No. 108), and L7 (Longshu No. 7) are relatively less susceptible than other main varieties. The pathogenicity index of SM on QS9 only reached 50% on the thirteenth day after infection. The pathogenicity index of SM on LS6 (Lishu No. 6), DF3 (Dafeng No. 3) and SY2 (Shiyan No. 2) reached 50% on the third day of infection (Supplementary Figure S1). Among them, JZ1 (Jingzhang No. 1) has the fastest onset, and the pathogenicity index of SM is greater than 60% on the third day of infection. However, the pathogenicity of DQ is very low among all varieties, with the pathogenicity index not exceeding 15%. For foreign varieties, the A292 variety had strong disease resistance, and the pathogenicity index of SM and DQ did not exceed 20% thirteenth days post inoculation (Figure 1E). In addition, the variety RA517-69 is the most susceptible to the disease, and the pathogenicity index of SM reached 50% 1 day post inoculation (Supplementary Figure S2). To rule out that this pathogenicity difference was due to the different growth rates of the bacteria, we measured the growth curves of the two bacterial strains (Supplementary Figure S3). The results showed that the growth rates of the two bacterial strains were similar, and both entered a growth plateau after 16 h. In summary, SM and DQ exhibit significant pathogenicity variation assayed on multiple potato varieties.

### Genome sequencing and phylogenetic analyses of *Pectobacterium brasiliense* SM and DQ

To gain a deep insight into the genome features of these two strains, we sequenced the entire genome of SM and DQ (Table 1). The genomes of SM and DQ consist of the single circular chromosome that are 4,953,627 and 4,764,461 bp in size with no plasmid. The average GC content in the whole genome of SM is 52.03%, which is similar to that of DQ (52.13%), SX309 (52.18%), and 1692 (52%). A total of 4,390 and 4,331 Open Reading Frames (ORFs) were predicted in the SM and DQ genomes, respectively (Table 1). In the genome of SM, in addition to 4,285 protein-coding genes (CDSs), the chromosome also contains 127 RNA genes, including 77 tRNA genes. In the genome of DQ, 4,227 protein-coding genes (CDSs) and 123 RNA genes containing 76 tRNA genes were predicted (Supplementary Table S1). Through analysis with the Pfam and SignalP databases, we classified 3,018 (67.77%), 2,984 (67.51%), 305 (6.85%) and 282 (6,38%) genes with different Pfam domains and signal peptides, respectively (Supplementary Table S1). Among them, 4,254 (95.53%) and 4,142 (93.71%) of the predicted genes of SM and DQ were assigned to KEGG categories, respectively (Supplementary Table S1).

**Table 1:** Genomic features of *Pectobacterium brasiliense* SM, DQ, SX309, and 1692.

Features	SM	DQ	SX309	1692
Size (bp)	4,953,627	4,764,461	4,966,299	4,851,982
G + C content (%)	52.03	52.13	52.18	52
Total gene number	4,390	4,331	4,455	4,325
Protein-coding genes	4,285	4,227	4,351	4,219
Transfer RNA	77	76	76	77

To determine the phylogenetic status of SM and DQ, the phylogenetic tree of *Pectobacterium* spp. and *Dickeya* spp. was constructed based on 3,860 bp concatenated sequences of *gapA*, *gyrA*, *icdA*, *mdh*, *proA* and *rpoS* using MEGA11 software with Maximum Likelihood method and Tamura-Nei model (Supplementary Figure S4). The bootstrap values of 1,000 replicates display the significance of each branch. The MLSA-based phylogenetic tree indicated that strains SM and DQ belong to *Pectobacterium brasiliense* and have close distance with SX309 and the model strain 1692 as the near neighbors.

### Comparative genomics analyses reveal distinct genome features of *Pectobacterium brasiliense* SM and DQ strains

**Figure 2:**
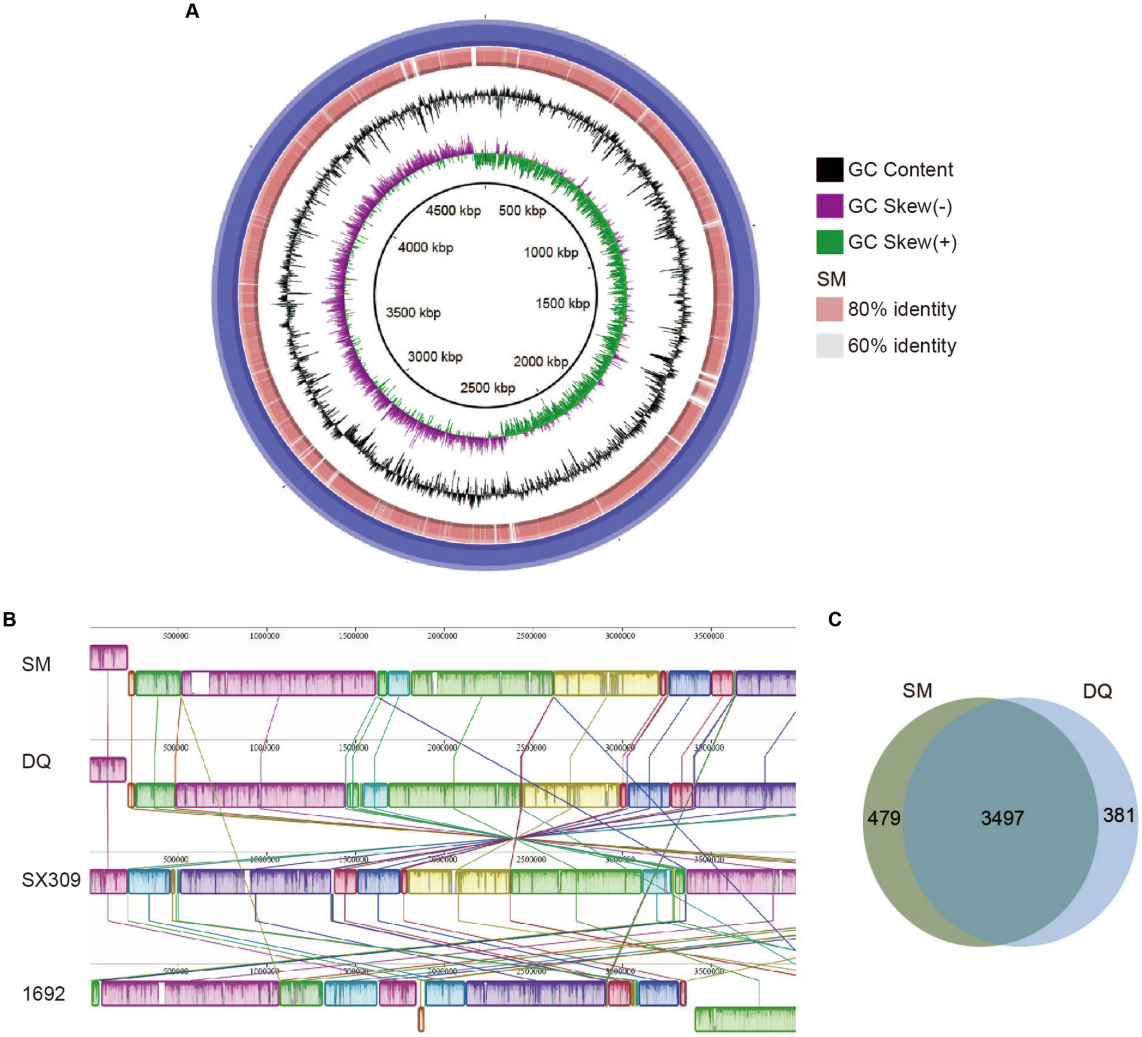
Comparison of *Pectobacterium brasiliense* SM and DQ genome sequences. **(A)** Circular map of genome comparison between SM and DQ. The first two rings correspond to GC skew and GC content, respectively. DQ is used as a reference genome shown as the outermost purple ring. The orange ring represents the results obtained after performing a BLAST analysis between SM and DQ. The light orange and gray areas denote distinct identity levels within the alignment. The identities are based on BLAST+ calculations. **(B)** Mauve progressive alignment of SM, DQ, SX309 and 1692 genomes, with SM genome serving as the reference. Collinear regions are represented by boxes of the same color. The box below the horizontal strain line denotes the inversion region, while rearrangements are indicated by colored lines. **(C)** Venn diagrams showing numbers of clusters of orthologous genes including strain specific genes and core genes.

To search for genetic features that might explain the virulence difference between these two strains, we performed comparative genomic analysis using the whole genome sequencing data. The genome alignment circular map was made with BLAST+ using BRIG (BLAST Ring Image Generator; Alikhan et al., 2011; Figure 2A). The map unveiled an exceptionally elevated level of homogeneity, exceeding 90% similarity between the two genomes. We calculated the ANI and DDH values of SM, DQ and 1692 (Supplementary Table S2). The findings indicate that the Average Nucleotide Identity (ANI) and DNA–DNA Hybridization (DDH) values between strains SM and DQ, when compared to each other, were approximately 97.27% and 86%, respectively. The DDH values calculated between strains SM and 1692, as well as between DQ and 1692, were found to be 79.2% and 80.5%, respectively. These values exceed the generally accepted species threshold of 70%, indicating a significant genetic relatedness between the strains. Additionally, the ANI values were 96.42% for SM and 96.09% for DQ compared to 1692, both clearly exceeding the threshold of 95% used for species demarcation. These results suggested that strains SM and DQ clustered closely and were at the same position in classification.

To assess the evolutionary distance between strains SM and DQ at the subspecies level, the whole genome sequences of SM and DQ were aligned with their closest sequenced relatives, SX309 and the type strain 1692 using Mauve (Figure 2B). The alignment revealed a high degree of similarity between the two genomes, with a pronounced collinearity. Notably, both SM and DQ genomes exhibited greater similarity to SX309 than to the type strain 1692. This observation supports the relationships elucidated in the phylogenetic analysis. In comparison to SX309 and 1692, there is no obvious gene inversion or deletion of large regions in SM and DQ genome, but a large area of local collinear block (LCB) inversion occurred. This pairwise comparison supports the analysis results that strains SM and DQ belong to the same subspecies.

In order to identify the specific genes with respect to pathogenicity in SM and DQ, we performed a pan-genomic comparative analysis of the two genomes (Figure 2C). The statistical analysis unveiled a comprehensive set of 4,357 pan-gene clusters, comprising 3,497 core orthologous gene clusters (80.26%) and 860 dispensable gene clusters (19.74%). Within the 860 dispensable gene clusters, 479 (10.99% of all pan gene groups) specific gene clusters unique to the SM genome were absent in the DQ genome. Conversely, the DQ genome harbors 381 unique gene clusters, and the functions of some of these unique genes remain currently unknown. Specific genes associated with KEGG functional categories were stated. Only SM has one specific gene in the category of cell motility, signal transduction, energy metabolism and nucleotide metabolism. The specific genes in SM and DQ strains are largely distributed in membrane transport and carbohydrate metabolism (Supplementary Table S3). In summary, the core-pan gene analysis indicated that the SM strain possesses a greater number of unique gene groups.

### Genes encoding plant cell wall-degrading enzymes and secretion systems exhibit great variations between SM and DQ

The production of plant cell wall-degrading enzymes and secretion of these enzymes are required for *Pectobacterium brasiliense* infection. To identify whether SM and DQ strains have different plant cell wall-degrading enzymes and secretion systems which are related to pathogenicity, we compared the plant cell wall degrading enzymes and secretion systems genes in SM and DQ. A total of 42 and 41 related genes encoding pectinases, cellulases, and proteases were identified in the SM and DQ genomes, respectively (Supplementary Table S4). Concisely, the SM genome encompasses 18 genes that encode pectin-degrading enzymes. Notably, these pectin degradation function genes are conserved in both the SM and DQ genomes. Likewise, 9 protease-encoding genes were identified in both the SM and DQ genomes. Furthermore, the SM genome features 15 genes associated with cellulose degradation, encompassing cellulases, endoglucanase, beta-glucosidase, and cellulose synthase operon proteins. Among these genes, a majority are conserved in both genomes. However, one gene encoding endoglucanase was found to be absent in DQ. This gene belongs to glycosyl hydrolase 12 family, and it functions in polysaccharide catabolic process which may contribute to pathogenicity. In general, there is little significant difference in the number of cell wall-degrading enzymes between the two bacteria except one endoglucanase S gene which is encoded specifically in SM.

**Figure 3:**
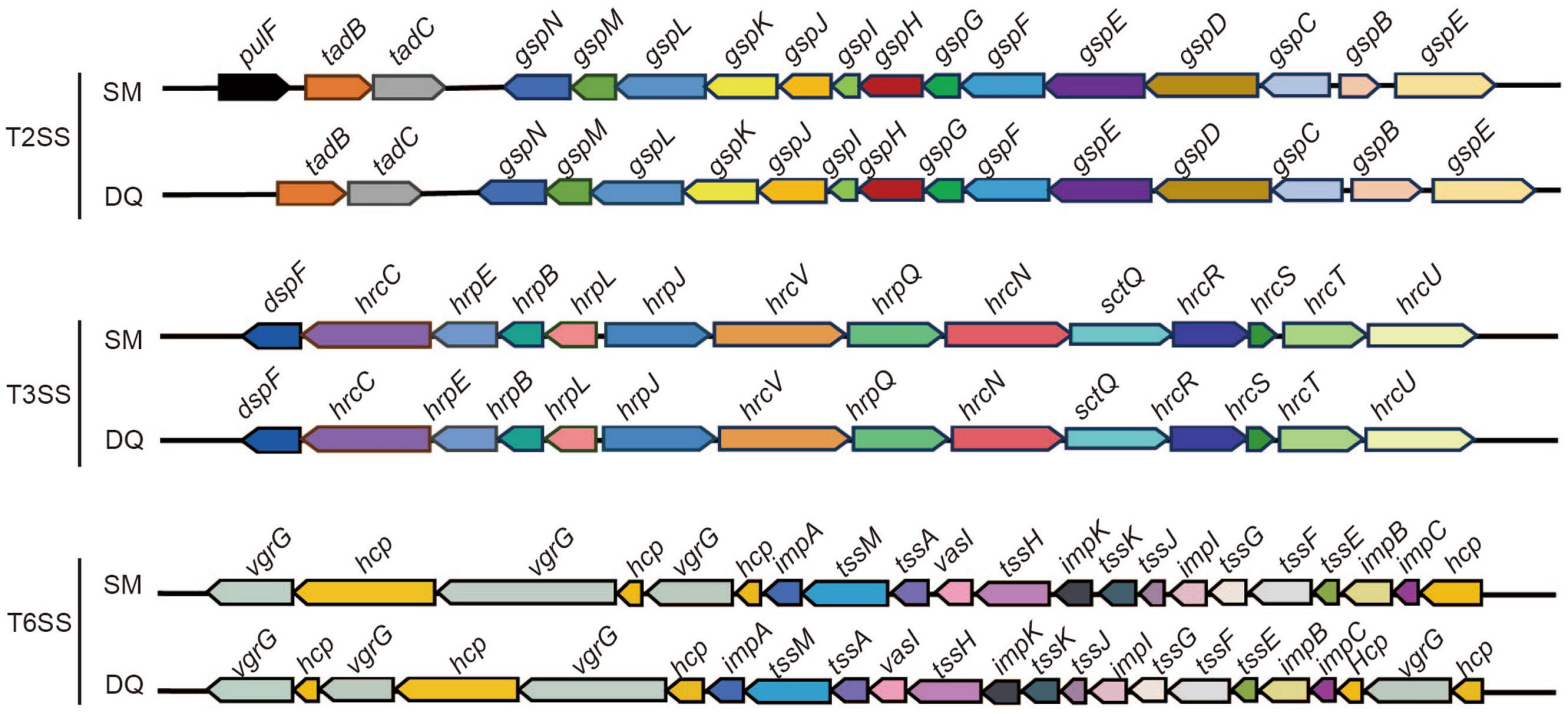
Illustration of gene organization of type II secretion system (T2SS), type III secretion system (T3SS) and type VI secretion system (T6SS) in *Pectobacterium brasiliense* SM and DQ. Arrows denote putative transcriptional units.

The SM and DQ genomes harbored multiple secretion systems, including the type II secretion system (T2SS), type III secretion system (T3SS), and type VI secretion system (T6SS), which were closely related to bacterial pathogenicity (Figure 3). Both SM and DQ lacked Type IV secretion system and Type V secretion system. SM had one gene *lapB* specifically for Type I secretion system. The T2SS genes were conserved between SM and DQ, except that *pulF* was absent in DQ. Many plant pathogenic bacteria employed T3SS to inject a diverse array of toxic proteins into plant cells to facilitate their infection. An *hrp/hrc* gene cluster containing 12 genes was found in the SM and DQ genomes, and these genes were highly conserved in the two genomes. T6SS was widespread in many Gram-negative bacteria and deliver toxic effector proteins to neighboring bacteria or host cells. In this study, the prevalence of T6SS was observed in both the SM and DQ genomes, with the SM genome containing 31 T6SS-associated genes and the DQ genome containing 29 such genes. There were 4 and 5 *vgr*G genes and 13 and 10 *hcp* genes in the genomes of SM and DQ, respectively. These genes encoded extracellular structural components of the secretion machinery and specific effectors (Supplementary Table S5). The copy number of *vgr*G and *hcp* genes appeared to be different between two subspecies. In summary, SM possesses more secretion system gene cluster genes compared to DQ. Notably, SM harbors two genes specifically related to type I secretion system and type II secretion system.

### Transcriptome analyses *in vivo* and *in vitro* unravel distinct gene expression patterns in *Pectobacterium brasiliense* SM and DQ

**Figure 4:**
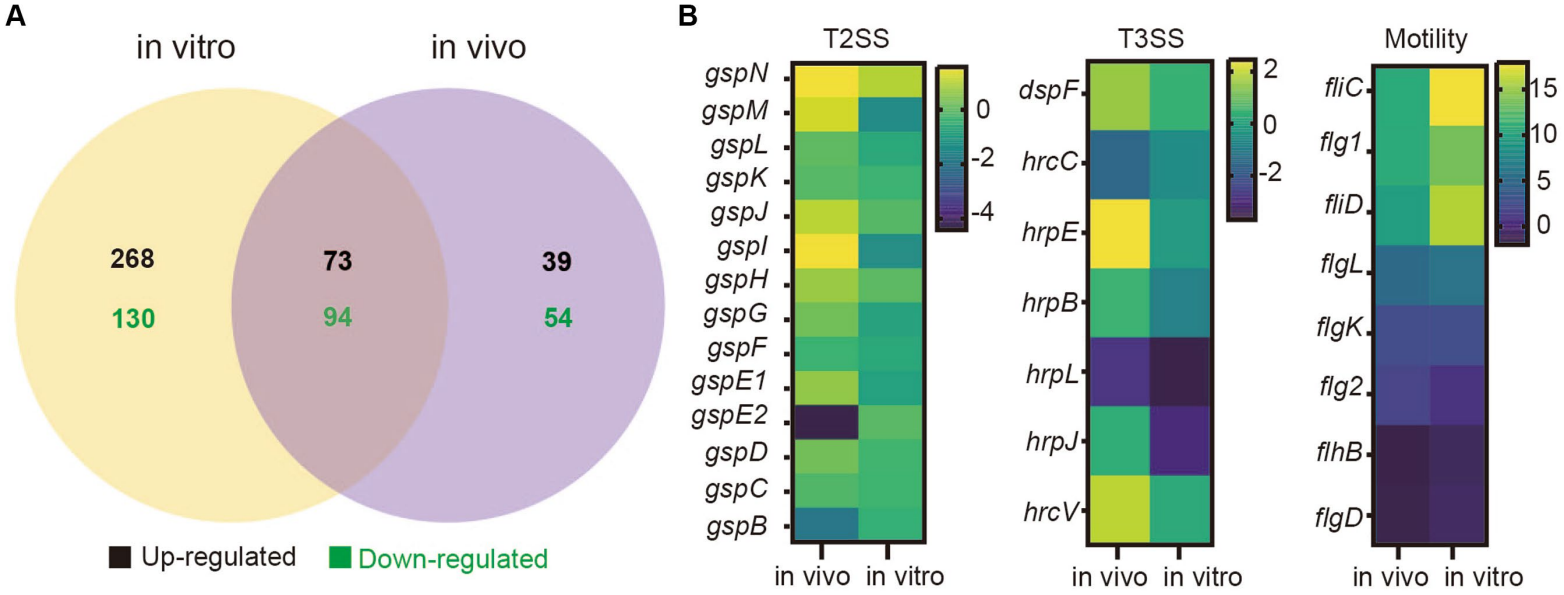
Transcriptome analyses of *Pectobacterium brasiliense* SM and DQ under *in vivo* and *in vitro* conditions. **(A)** The number of different expressed genes (SM vs. DQ) in nutrient broth medium (*in vitro*) and potato host (*in vivo*). **(B)** Transcriptional variation profiles of T2SS, T3SS cluster genes and flagellar assembly genes comparing SM to DQ under two conditions. Each row on the heatmap represents a gene, labeled accordingly. The variation in transcription illustrated by a color scale representing the log2 (FPKM).

To determine the differences in gene expression between SM and DQ strains, transcriptome sequencing was performed for two strains under two distinct conditions: cell grown in nutrient broth (*in vitro*) and cells recovered from infected potato stems (*in vivo*). RNA-Seq analysis was validated by qRT-PCR with three biological replicates (Supplementary Figure S5). Our analysis revealed distinct gene expression patterns in both strains under different conditions. In SM, a comparison between *in vivo* and *in vitro* conditions unveiled 951 upregulated and 758 downregulated genes (Supplementary Table S6). Similarly, DQ exhibited 940 upregulated and 837 downregulated genes in the potato host environment compared to *in vitro* culture (Supplementary Table S6). Moreover, a comparison of conserved genes between SM and DQ showed differential expression patterns (Figure 4A). Under *in vitro* conditions, 565 genes displayed differential expression, with 341 genes upregulated and 224 genes downregulated. In the potato host environment, 260 genes exhibited significant differential expression, comprising 112 upregulated and 148 downregulated genes (Supplementary Table S6). Among these, 167 differentially expressed genes were found to overlap between the two conditions, with 73 upregulated and 94 downregulated genes. This suggests that the expression levels of these genes were constitutively different and independent of growth conditions. We observed a large number of strain-specific different expressed genes under *in vitro* conditions, while a small portion of strain-specific genes changed their expression in host environment (Figure 4A). This observation implied that biochemical and physiological activities of these two strains were distinct when grown in NB medium, although they were phylogenetically similar. Conversely, when infecting plants, these two strains tend to employ similar virulent mechanisms or strategies for host colonization.

Comparing to DQ strain, SM exhibited significant upregulation of T2SS related genes in plant host (Figure 4B). Similar results were also observed for T3SS genes (Figure 4B). Genes related to bacterial motility were consistently upregulated in both conditions, but more significant *in vitro*, in which genes, such as *fli*C, *flg*1, *fli*D and *flg*L, exhibited the higher expression levels in NB medium compared to plant host (Figure 4B). Additionally, KEGG pathway analysis revealed that flagellar associated genes were enriched (Supplementary Figure S6). Taken together, our data suggest that SM strain has higher expression of genes related to T2SS, T3SS and motility, which are important virulence traits for bacterial infection.

### *Pectobacterium brasiliense* SM exhibits higher motility and pectinase, cellulase, and protease activities than DQ strain

**Figure 5:**
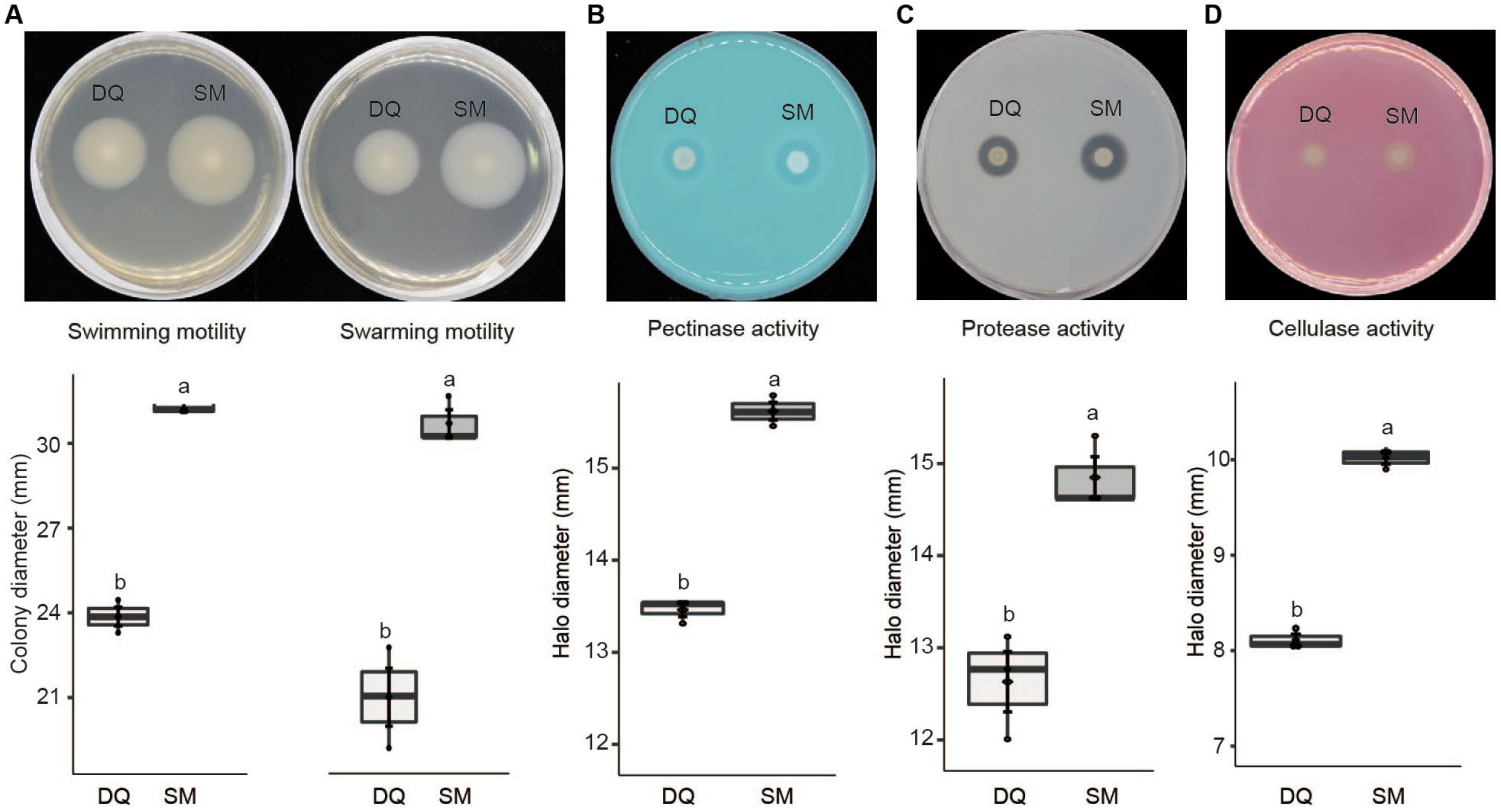
Phenotypic characteristics of *Pectobacterium brasiliense* SM and DQ strains. **(A)** Swimming and swarming motility was estimated by the diameter (mm) of the spreading of the colonies. **(B)** Pectinase activity in the medium with PGA was estimated by the diameter (mm) of the haloes observed on the detection plates. **(C)** Protease production was estimated by the halo diameter (mm) observed on the assay plate. **(D)** Cellulase production was estimated by the halo diameter (mm) observed on the assay plate. All experiments were performed three times. The top and bottom lines of the boxplot refer to upper and lower quartiles, respectively. The ends of the whiskers reach minimum and maximum values. The dark diamond represents the mean, and the black bar depicts the median. Means ± standard errors are depicted (*n* = 3). Within each box, mean values marked with different letters are significantly different by One Way ANOVA analysis and Fisher’s least significant difference criterion (R agricolae package).

Given the fact that the flagellar assembly pathway in SM was enriched in transcriptome analysis, we conducted an assay to compare swimming and swarming motility of two strains on NB plates. The results indicated that SM exhibited greater swimming and swarming motility compared to DQ, which is consistent with our transcriptome data (Figure 5A).

Similarly, T2SS upregulation may also increase the secretion of pectinases, cellulases, and proteases, which are critical enzymes for *Pectobacterium* pathogenicity. To test this hypothesis, we evaluated these enzyme activities on the assay plates. The results indicated that the high virulent strain SM exhibited higher activity of pectinase (Figure 5B), protease (Figure 5C), and cellulase (Figure 5D). In summary, SM exhibits greater swimming and swarming motility, higher activities of PCWDEs than DQ, which explains the higher virulence of SM strain.

### SM-specific endoglucanase S promotes virulence of DQ

**Figure 6:**
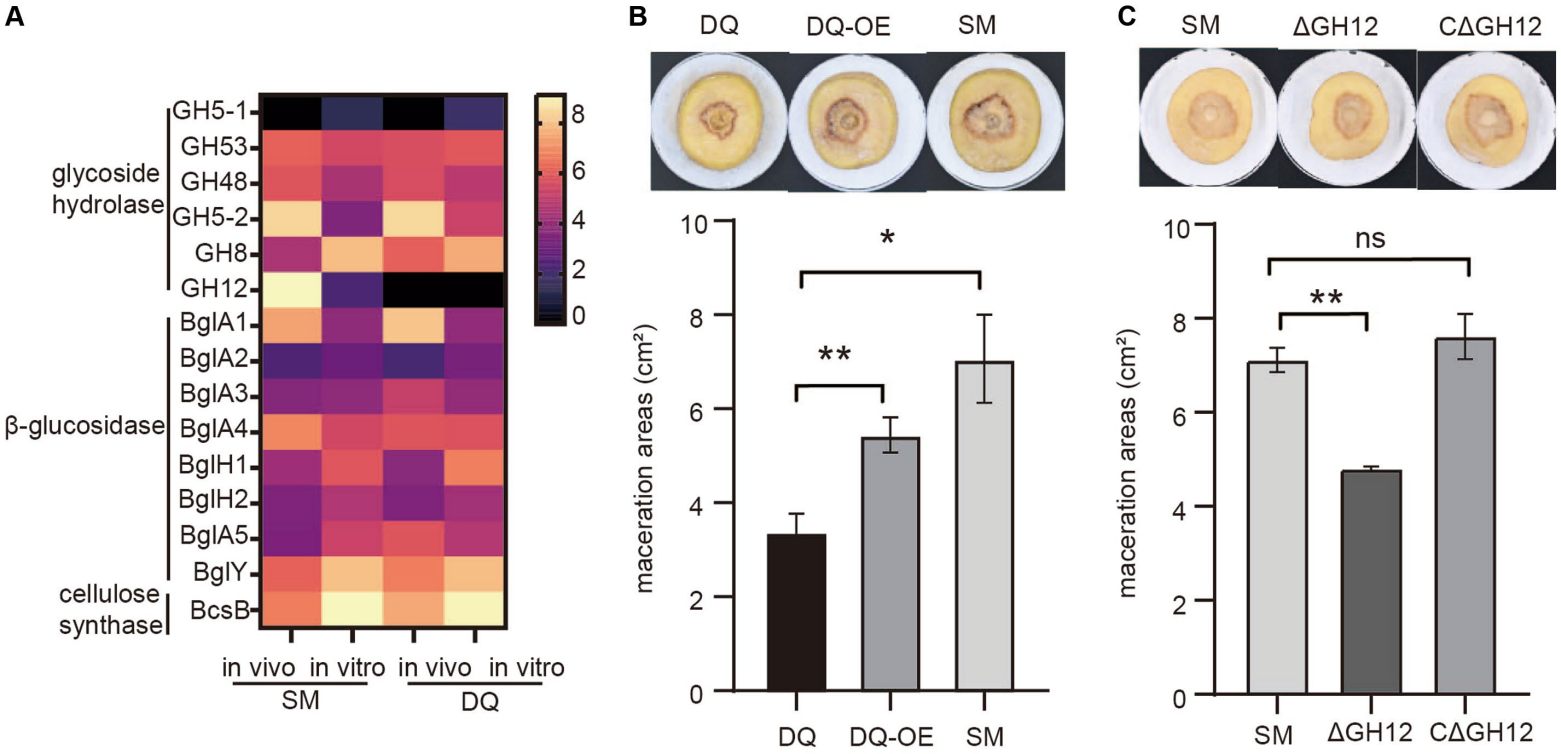
Validation of the pathogenic function of *Pectobacterium brasiliense* SM specific endoglucanase S gene GH12. **(A)** The heatmap illustrates the transcriptional variation profiles of cellulase including glycoside hydrolase, β-glucosidase and cellulose synthase genes in SM and DQ strains under plant and NB conditions, respectively. **(B)** Potato soft rot assay. The endoglucanase S gene (GH12) is overexpressed in DQ strains. The pathogenicity was compared to wild type DQ strain and SM strain. **(C)** Comparison of the potato slices maceration ability of the tested wild-type SM strain, GH12 knock-out (ΔGH12) SM strain and GH12-complemented expression strain (CΔGH12). Maceration areas of potato slices were measured after 24 h of infection.

To investigate the role of the cellulase in bacterial pathogenicity, we analyzed transcription levels of cellulase genes including glycoside hydrolases, β-glucosidases and cellulose synthases in both strains under *in vivo* and *in vitro* conditions. The heatmap showed that the expressions of most glycoside hydrolases were induced in plant in both strains, except GH5-1 and GH8. It is noteworthy that the endoglucanase S (GH12) gene, which was absent in DQ strain, exhibited significant high expression level in SM strain *in vitro*, with a fold change level exceeding 6 (Figure 6A). This result indicated that endoglucanase S (GH12) may play a crucial role in the process of bacterial infection and be a factor that determined the higher pathogenicity in SM.

To test this hypothesis, we cloned GH12 with its own promoter into a multi-copy plasmid to overexpress GH12 in DQ. The potato slices maceration assay demonstrated that DQ overexpressing GH12 (GH12OE) had higher pathogenicity compared to the wild type strain (Figure 6B). However, GH12OE failed to fully complement the virulence to SM level, implying that other SM specific factors also contributed to its virulence. To further elucidate the functional properties of GH12, we constructed knock-out and complementary expression strains for the GH12 gene and proceeded to evaluate their pathogenicity. The potato slices inoculation assay demonstrated that, compared to potato slices inoculated with the wild-type SM strain and those with the GH12-complemented expression strain (CΔGH12), that inoculated with the GH12 knock-out (ΔGH12) SM strain exhibited smaller lesion areas following maceration (Figure 6C). This observation suggests that the absence of the GH12 gene results in a reduced pathogenicity of SM. Taken together, we show that endoglucanase GH12, which is absent in DQ, significantly contributes to bacterial pathogenicity, likely via accelerated plant cell wall degradation.

## Discussion

*Pectobacterium brasiliense* is considered to be the most virulent species in the *Pectobacteriaceae* (Oulghazi et al., 2021). In this study, genomes and transcriptomes of two *Pectobacterium brasiliense* strains SM and DQ, which were isolated from our lab, were explored to disclose some explanation for their diverse ability to macerate plant tissue. SM and DQ were assigned to the clade of *Pectobacterium brasiliense* with the other reported strains (SX309 and 1692) based on the phylogenetic analysis. Furthermore, ANI and DDH values show the classification position of SM and DQ. Pan-genome analysis provides an effective method for characterizing bacterial composition difference and elucidating variations in strain pathogenicity. Genes found in dispensable genome segments may be responsible for the ability of a strain to survive in rare or specific conditions (Golanowska et al., 2018). Additionally, since these two strains were isolated from regions with significantly different climates in the northern and southern areas, we speculate that their varying pathogenicity may be associated with the different stress resistance, which helps bacterial strains to deal with environmental cues. Further experiments are needed to quantify bacterial growth in different stress conditions.

Gene redundancy serves as a protective strategy for the bacteria to mitigate the impact of adverse changes in the external environment on their survival. Comparative analysis of SM and DQ strains identified multiple function redundant strain-specific genes that might contribute to the differences in virulence and host adaptability. SM genome has two more *fhaB* adhesin genes than that of DQ. The *fha* gene encodes a large filamentous hemagglutinin secreted from cells. These Fha proteins promote the attachment of bacterial cells to the host. Studies have shown that FhaB proteins have similar domain structures. The most variable region found in CdiA/FhaB is the cytotoxicity domain following a common pre-toxin VENN motif (Golanowska et al., 2018). These proteins are highly variable, we need to further investigate the phylogenetic relationships of the cytotoxic domains extracted from adhesion proteins of SM and DQ to identify their function on the pathogenicity. Furthermore, KEGG pathway analysis of the unique genes specific to the SM strain revealed that SM has one specific gene in the category of cell motility, signal transduction, energy metabolism and nucleotide metabolism. Bacteria employ a variety of strategies to interact both with communication and the environment. Mechanisms using diffusible signaling molecules plays a role in bacterial virulence. Chemotaxis represents the guided movement strategy employed by microorganisms to perceive chemical stimuli and subsequently navigate toward more advantageous environmental conditions (Salah Ud-Din and Roujeinikova, 2017). SM has one specific gene coding Methyl-accepting chemotaxis protein (MCP). MCPs play an important role in cellular endurance, disease-causing potential, and biodegradation capabilities. A variety of MCP classes have been recognized based on the distinguishing characteristics of their ligand-binding domains and membrane topology. In future studies, there is potential to delve deeper into the classification and pathogenic function of the specific MCP present in the SM strain. These potentially virulence-enhancing specific genes are promising targets for drug development, thereby exerting detrimental effects on bacterial survival and pathogenicity, ultimately enhancing the host’s ability to eliminate infection.

PCWDEs, including pectinases, cellulases and proteases, are key virulence factors for many important plant bacterial pathogens causing soft rot disease (Joshi et al., 2015). In this study, SM has one specific cellulase gene endoglucanase S which is highly upregulated in SM recovered from plants compared to in NB condition. Expressing the endoglucanase S gene in DQ significantly enhances the soft rot pathogenicity of DQ (Figure 6). The endoglucanase S gene belongs to the GH12 family (glycoside hydrolase family 12). GH12 enzymes can collaborate with other cellulases to enhance the efficiency of lignocellulosic biomass conversion (Zhu et al., 2019). Cellulases belonging to GH families function through an acid–base catalytic mechanism, often involving inversion of configuration at the anomeric center or retention (Ibrahim et al., 2017). The GH-12 family features two Glu residues situated on opposite sides of the substrate-binding cleft, serving as the catalytic nucleophile and the Brønsted acid/base. This arrangement facilitates their catalytic actions through a double displacement, retention mechanism (Zechel et al., 1998). This kind of endocellulase was first discovered from a cellulolytic actinomycete, *Streptomyces* sp. LX. It produces an extracellular cellulase with high CMC liquefaction activity and acetylase activity, responsible for the cleavage of cellulose (Li et al., 1998). This warrants further exploration of the significance of endoglucanase S in the bacterial infection process. Additionally, purifying this protein and investigating its potential applications would be valuable.

T3SS is used by many Gram-negative pathogenic bacteria to deliver virulence proteins, or known as type III effectors, into host cells. Once inside the host cell, effectors manipulate host defenses and promote bacterial growth (Grant et al., 2006). In the part of transcriptome sequencing analysis, we found 5 SM-specific effectors overexpressed in host plant. Among which, GM004001 was identified having the ability to induce cell death in tobacco post 4 days inoculation (Supplementary Figure S7). The subcellular localization of the effector protein indicates that it is localized in the cell nucleus. Subcellular localization prediction has also shown it is localized in nucleus by Plant-mPLoc (Chou and Shen, 2010). Alfano and Collmer (2004) proved that T3SS effectors, such as AvrBs3 family members, may act in the nucleus to alter host transcription. The mechanisms of nuclear import may vary among effectors. For example, AvrBs3 family members carry nuclear localization signals (NLS; Van Den Ackerveken et al., 1996). In contrast, the PopP2 effector appears to help direct the R protein RRS-1 to the nucleus, as RRS-1 localizes to the nucleus only in the presence of PopP2 (Deslandes et al., 2003). Determining the localization of other T3SS effectors in plant cells is another systematic approach to exploring their functions. Besides, tertiary structure prediction showed the highest similarity model (96.95%) of the protein is the toxic anion resistance protein in *Pectobacterium versatile* by using SWISS-MODEL (Waterhouse et al., 2018). The function of this protein deserves to be further studied.

Bacterial flagella are intricate organelles with origins traceable to early cellular structures. They facilitate swimming and swarming motility and play pivotal roles in adhesion, biofilm formation, and host invasion (Liu and Ochman, 2007). Inactivation of the *flgA*, *fla* and *flhB* genes inhibited the bacterial viability and significantly reduced the virulence of the *Pectobacterium carotovorum* PCC21 (Lee et al., 2013). We observed that the SM strain with strong pathogenicity exhibited higher motility and expression of flagellar related genes during the plant infection process. Motility is likely coordinated with the production of plant cell wall-degrading enzymes (PCWDE) and the secretion system responsible for virulence. The specific role of flagella genes in *Pectobacterium* pathogenicity, particularly in interactions with host plants, warrants further exploration.

In summary, a comparative genomic analysis of the highly virulent *Pectobacterium brasiliense* SM and the low virulent DQ strains revealed minimal genetic differences. However, notable distinctions were observed in the production of virulence factors, including pectinases, cellulases, and proteases, as well as in their motility. This study offers insights into the virulence mechanism of this significant pathogenic bacterium. Additionally, we conducted a comparison of gene expression profiles between *Pectobacterium brasiliense* SM and DQ in nutrient broth (NB) and potato host. Our findings revealed the induction of virulence genes, including those encoding T2SS and motility-related genes during the infection process. Numerous genes with differential expression levels between SM and DQ strains were identified. In particular, the cellulase endoglucanase S, which is absent in DQ, exhibited significantly high expression during the infection process in SM. We experimentally verified the role of this endoglucanase S in bacterial virulence. This study provides new insight into the phenotypic features and potential virulence determinants of *Pectobacterium brasiliense.*

## Materials and methods

### Bacterial strains, primers, plasmids, and plant materials

Bacterial strains, primers, plasmids and gene sequences are listed in Supplementary Tables S7, S8. *Pectobacterium brasiliense* SM and DQ were isolated from typical blackleg symptomatic potato stems in Sanming, Fujian Province, and Erdos, Inner Mongolia, respectively, in 2019. These strains are routinely cultured in Difco Nutrient Broth (NB) medium or on Difco Nutrient Agar (NA) at a temperature of 30°C. *Escherichia coli* DH5α carrying pH2-GH12 plasmid was cultured at 37°C in Luria Broth medium. Kanamycin (50 μg mL^−1^) was supplemented when needed. Strains were stored in the medium supplemented with 50% glycerol at −80°C.

### Pathogenicity assay

Three inoculation approaches are conducted in our study. For aerial stem rot, 100 μL of bacterial suspension (10^8^ CFU/mL) was injected into the stem base of potato plants. Inoculated plants were placed in a greenhouse at 21°C with 80% humidity. The plant phenotypes were observed and photographed continuously for 1 week after infection for the calculation of pathogenicity index. The pathogenicity assay of potato blackleg was conducted through a bacterial soil drench experiment. Each pot was filled with 500 mL of bacterial suspension (10^8^ CFU/mL) and the incidence rate was monitored over a two-week period. For potato chip experiment, potato tubers were washed in clean water, rinsed in alcohol, and then sliced and dried on a clean bench. Three potato chips in each replicate were inoculated with 100 μL of bacterial suspension (OD600 = 0.1 or 0.3) by pipetting and incubated at 30°C for 24 h. Maceration area of the lesions were measured using ImageJ. All experiments were conducted in three replicates.

### Phylogenetic relationship

Multilocus sequence analysis (MLSA) was used to conduct phylogenetic relationships among 6 housekeeping genes including *gapA*, *gyrA*, *icdA*, *mdh*, *proA* and *rpoS* from 16 strains of *Pectobacterium* spp. and 8 strains of *Dickeya* spp. Amino acid sequences were aligned using MUSCLE. Subsequently, the six housekeeping gene sequences were concatenated in the same order. The evolutionary history was inferred by using the Maximum Likelihood method from MEGA11 software (Tamura et al., 2021), and 1,000 bootstrap replicates were included in a heuristic search by applying Neighbor-Join and BioNJ algorithms to a matrix of pairwise distances estimated by Tamura-Nei model (Ortí et al., 1997).

### Whole-genome sequencing, assembly and gene prediction

The genomic DNA was extracted using the SDS method (Lim et al., 2016). The extracted DNA was assessed via agarose gel electrophoresis and quantified using the Qubit® 2.0 Fluorometer (Thermo Scientific). The complete genome of *Pectobacterium brasiliense* SM and *Pectobacterium brasiliense* DQ was sequenced at Beijing Novogene Bioinformatics Technology Co., Ltd. using PacBio Sequel platform and Illumina NovaSeq PE150. Single Molecule Real-Time (SMRT) sequencing library was constructed using SMRT bell TM Template kit, version 1.0, with an insert length of 10 kb. Long reads which longer than 6000 bp, were selected as seed sequences using the automatic error correction function in the SMRT portal, while the remaining shorter reads were aligned to these seed sequences using Blasr. The preliminary assembly results were further refined using the Variant Caller module in the SMRT Link software. The corrected assembly result serves as a reference sequence, which is then aligned with Illumina data using Burrows-Wheeler Aligner (BWA). The circularity of the chromosomal sequence was verified by assessing the overlap between the head and the tail. Then the initial site was further refined through BLAST with the DNAa database. Coding genes were retrieved using GeneMarkS program. tRNA genes were identified using tRNAscan (Lowe and Chan, 2016). Genes functions were predicted based on a BLASTP search against the Non-Redundant Protein Database. KEGG annotation results were obtained using the Diamond software. Furthermore, secretory proteins and secretion systems proteins were predicted using SignalP database (Bendtsen et al., 2004) and Swiss-Prot (The UniProt Consortium, 2023), respectively.

### Comparative genome analysis

Average Nucleotide Identity (ANI) values were calculated for the genomes using online Average Nucleotide Identity Calculator (EzBioCloud; Yoon et al., 2017). *In silico* DNA–DNA hybridization (DDH) was calculated using the Genome-to-Genome Distance Calculator (GGDC; Auch et al., 2010). The synteny analysis results of the genomic sequence were obtained by Mauve (Darling et al., 2004) using SM genome as the reference genome. The genome alignments were performed with BLAST+ using BRIG (Alikhan et al., 2011). Orthology assessment was conducted through CD-HIT rapid clustering of similar proteins to obtain core genes and specific genes. Each annotated gene is classified into a pan-genome structure, including core (present in all genomes analyzed), or unique (present in only one genome) pan-genome fragment.

### Transcriptome sequencing and analysis

Total RNA samples were quantified using spectrophotometry. RNA quality assessment was performed using the Agilent 2,100 Bioanalyzer (Agilent Technologies, CA, United States) at Beijing Novogene Bioinformatics Technology Co., Ltd. To remove rRNA and purify mRNA from total RNA, probes were employed. Initially, cDNA was synthesized using random hexamer primers and M-MuLV Reverse Transcriptase. DNA polymerase I and RNase H were used to synthesize the second strand of cDNA. PCR was then performed using Phusion High-Fidelity DNA polymerase, Universal PCR primers, and Index primers. Subsequently, PCR products were purified using AMPure XP beads, and the library quality was assessed using the Agilent Bioanalyzer 2100 system. After cluster generation, libraries were prepared and sequenced on the Illumina Novaseq platform, generating 150 bp paired-end reads. The FPKM of each gene is calculated based on the length of the gene and the number of reads of the gene (Mortazavi et al., 2008). Differential expression analysis was performed using the DESeq R software package (1.18.0). The resulting *p*-values were adjusted using Benjamini and Hochberg’s method to control the false discovery rate (Benjamini and Hochberg, 1995). KOBAS software was utilized to identify the statistical enrichment of differentially expressed genes in the KEGG pathway.

### Bacterial motility and enzymes activity assay

Swimming and swarming motility were tested by inoculating bacterial suspension on nutrient agar plates solidified with 0.3 and 0.6% agar, respectively. The colony diameter was measured after 24 h of incubation. Detection of pectinase activity was assayed on M63 polygalactonate (PGA) plate. After incubation for 48 h, the culture medium was stained with 10% copper acetate aqueous solution. Degradation of PGA results in the appearance of a white halo around bacterial colonies, which is measured to assess total pectinase activity. The ability to produce cellulases was analyzed on M63 with 0.2% glycerol and 1% carboxymethylcellulose. After incubation for 48 h, the plates were flooded with 0.1% Congo red solution for 10 min and then washed with 1 M NaCl for 5 min. The diameter of the halo that appeared around the colonies indicated cellulase activity and was subsequently measured. Determination of protease activity on medium containing 1% skim milk. After 24 h of incubation, measure the diameter of the transparent halo around the colony, reflecting protease activity. All the plate assays mentioned above were inoculated with 2 μL of bacterial suspension containing 10^8^ CFU ml^−1^. All experiments were performed three times with three replicates.

## Data availability statement

The Complete Genome sequences of SM and DQ have been deposited at GenBank under the BioProject PRJNA1018677 (https://www.ncbi.nlm.nih.gov/bioproject/PRJNA1018677/) and PRJNA1018699 (https://www.ncbi.nlm.nih.gov/bioproject/PRJNA1018699/). All other data are available on request from the author.

## Author contributions

YSu: Formal analysis, Investigation, Writing – original draft, Writing – review & editing. HU: Supervision, Validation, Visualization, Writing – review & editing. YW: Investigation, Writing – review & editing. QS: Investigation, Writing – review & editing. ZF: Investigation, Writing – review & editing. YSh: Validation, Writing – review & editing. RZ: Funding acquisition, Writing – review & editing. XZ: Conceptualization, Data curation, Funding acquisition, Project administration, Resources, Supervision, Writing – original draft, Writing – review & editing. JW: Conceptualization, Data curation, Formal analysis, Funding acquisition, Investigation, Methodology, Project administration, Supervision, Writing – original draft, Writing – review & editing.

## Supplementary material

SUPPLEMENTARY FIGURE S1 The disease index of SM and DQ were assessed on major domestic cultivated varieties, including QS9, LS6, L7, DF3, SY1, JZ1, YS108, and SY2. The evaluation was conducted over a 13-day infection period, and visual comparisons, supported by images, were made on the 13th day post-inoculation.

SUPPLEMENTARY FIGURE S2 Assessment of the disease index caused by SM and DQ on foreign resource varieties, including 398160.105, AY, A79, A292, R91121-16, and RA517-69, over a 13-day infection period. Comparative images were captured on the 13th day post-inoculation.

SUPPLEMENTARY FIGURE S3 Growth curves depicting the progression of SM and DQ in NB (Nutrient Broth). The values at each time point represent the means of three replicates, with Means ± SD presented in the plot.

SUPPLEMENTARY FIGURE S4 The phylogenetic tree of *Pectobacterium* spp. and *Dickeya* spp. was constructed based on 3860 bp concatenated sequences of *gapA*, *gyrA*, *icdA*, *mdh*, *proA* and *rpoS* using MEGA11 software with Maximum Likelihood method and Tamura-Neimodel. Asterisk indicates the strains used in this study. “(T)” indicates the type strains.

SUPPLEMENTARY FIGURE S5 qRT-PCR was employed to validate the RNA-Seq data. Six genes exhibited differential expression under the conditions in vivo and in vitro were selected respectively in SM and DQ. **(A)** Validation of gene expression levels in SM. **(B)** Validation of gene expression levels in DQ.

SUPPLEMENTARY FIGURE S6 KEGG enrichment analyses. **(A)** KEGG enrichment analysis of differentially expressed genes of SM compared to DQ recovered from potato stems after 24h infection. **(B)** KEGG enrichment analysis of differentially expressed genes of SM compared to DQ in NB medium.

SUPPLEMENTARY FIGURE S7 Heterologously expressed GM4001 causes cell death and subcellular localization of GM4001. **(A)** *Agrobacterium* mediated transformation of tobacco leaves for expression of GM4001 gene. **(B)** Subcellular localization of GM4001.
