# Supplementary figures and images for: Comparative genomic and transcriptome analyses of two Pectobacterium brasiliense strains revealed distinct virulence determinants and phenotypic features
Source: Front Microbiol. 2024 May 10;15:1362283. doi: 10.3389/fmicb.2024.1362283 (PMC11116658; doi:10.3389/fmicb.2024.1362283)

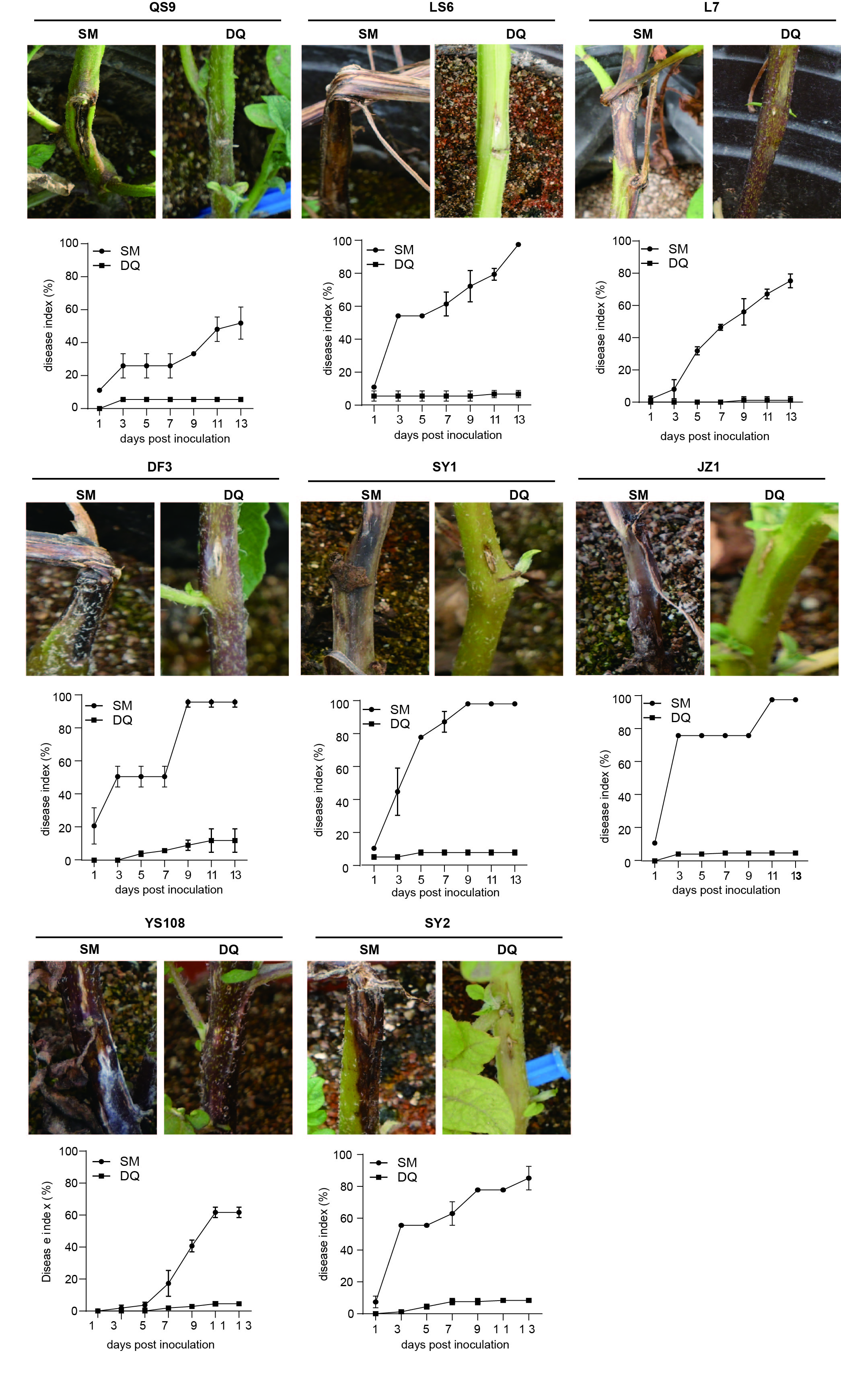

Supplement: SUPPLEMENTARY FIGURE S1 — The disease index of SM and DQ were assessed on major domestic cultivated varieties, including QS9, LS6, L7, DF3, SY1, JZ1, YS108, and SY2. The evaluation was conducted over a 13-day infection period, and visual comparisons, supported by images, were made on the 13th day post-inoculation. [file Image_1.JPEG]

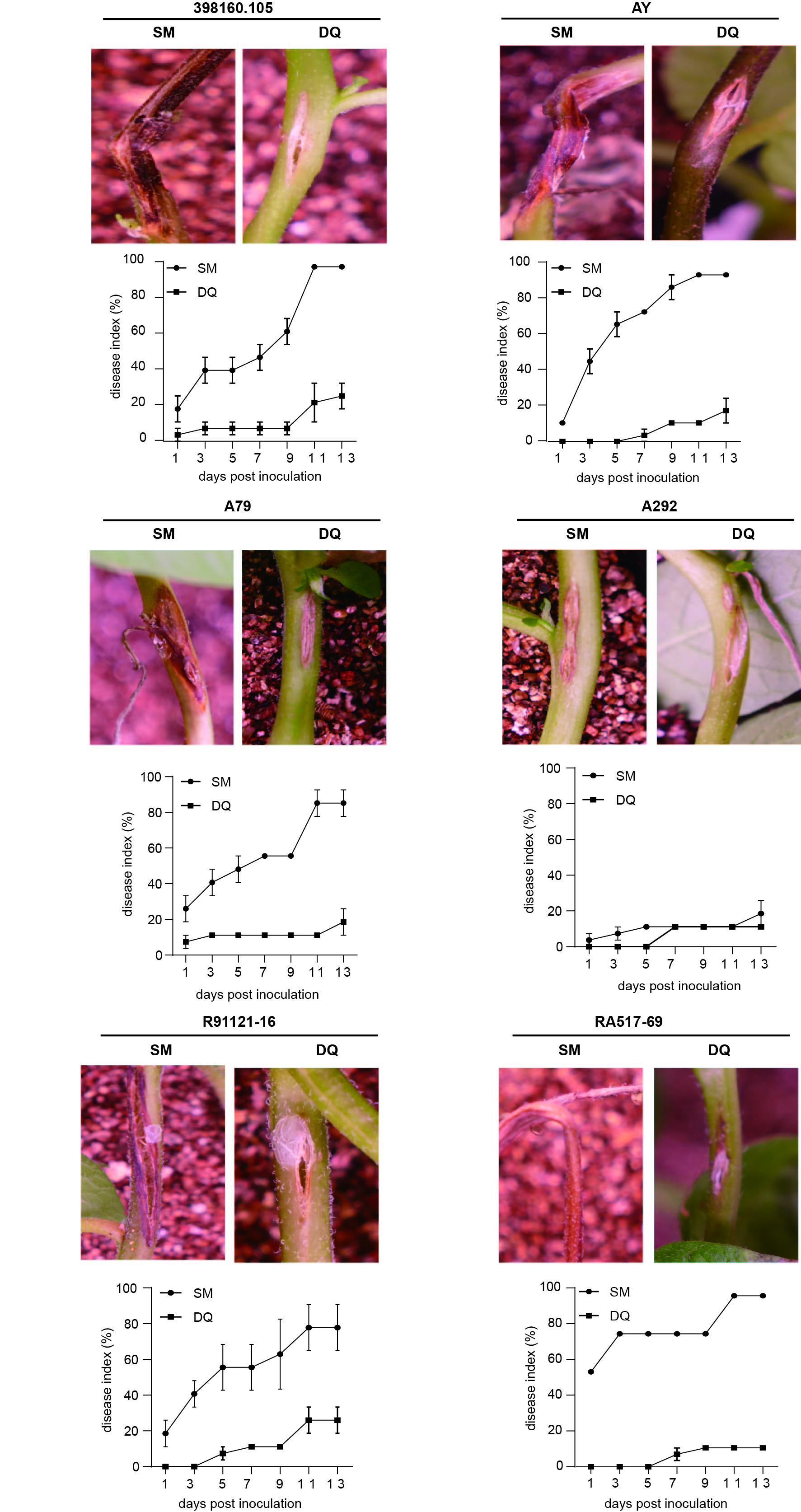

Supplement: SUPPLEMENTARY FIGURE S2 — Assessment of the disease index caused by SM and DQ on foreign resource varieties, including 398160.105, AY, A79, A292, R91121-16, and RA517-69, over a 13-day infection period. Comparative images were captured on the 13th day post-inoculation. [file Image_2.JPEG]

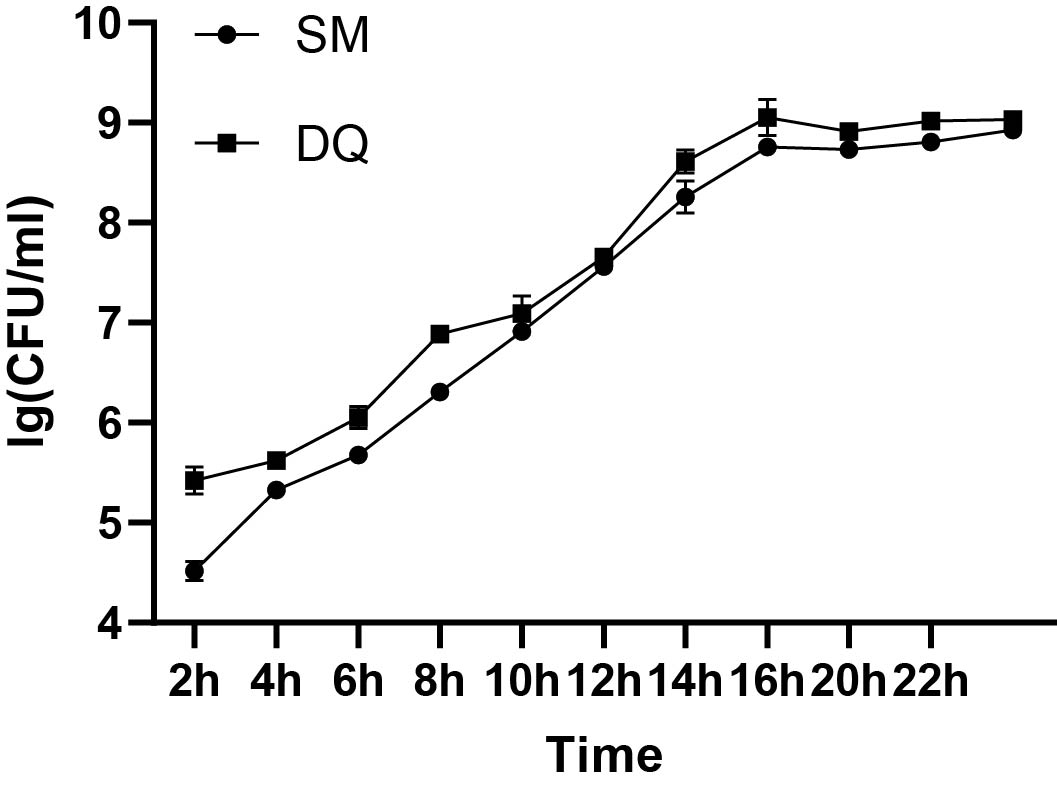

Supplement: SUPPLEMENTARY FIGURE S3 — Growth curves depicting the progression of SM and DQ in NB (Nutrient Broth). The values at each time point represent the means of three replicates, with Means ± SD presented in the plot. [file Image_3.JPEG]

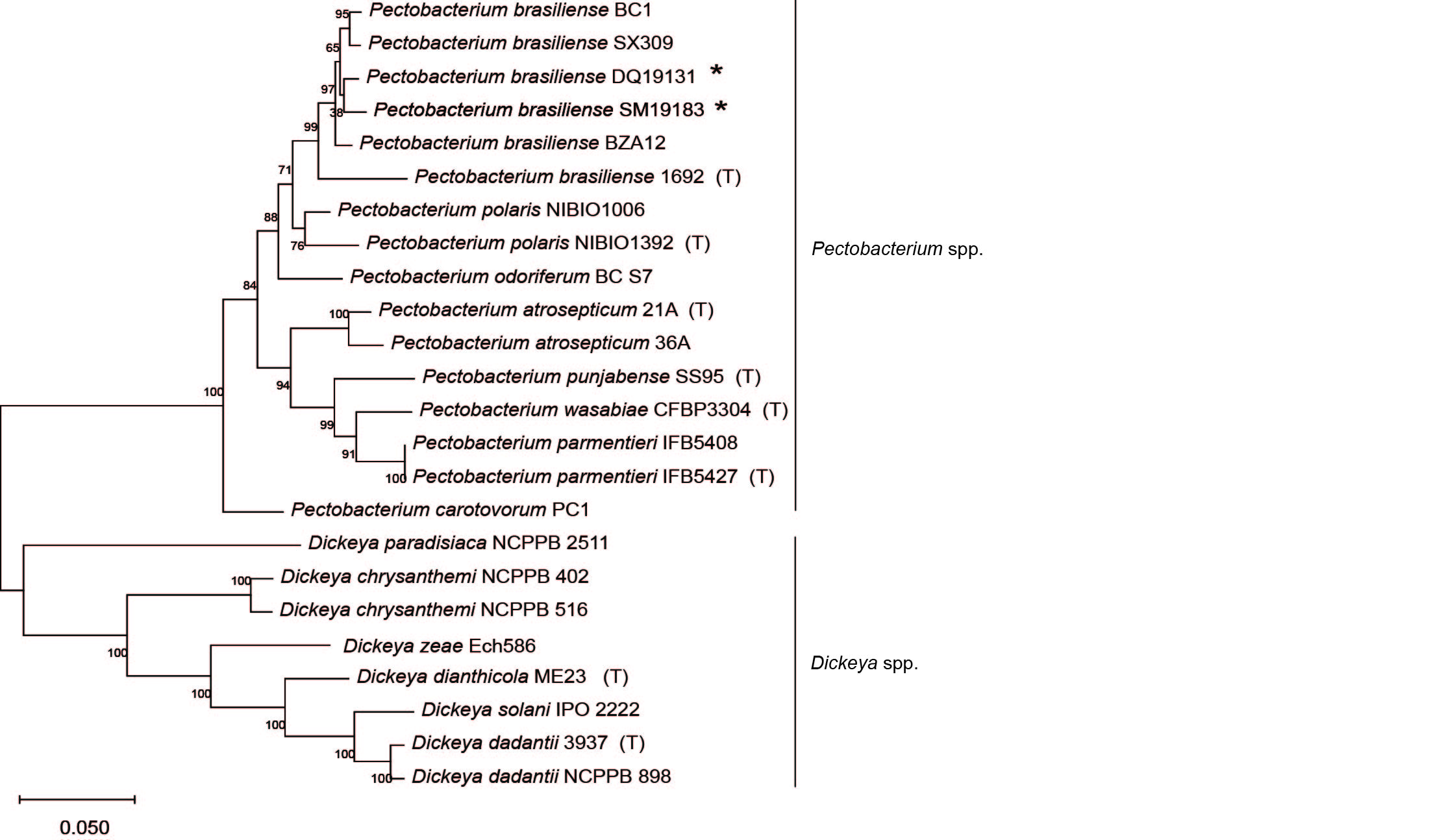

Supplement: SUPPLEMENTARY FIGURE S4 — The phylogenetic tree of Pectobacterium spp. and Dickeya spp. was constructed based on 3860 bp concatenated sequences of gapA, gyrA, icdA, mdh, proA and rpoS using MEGA11 software with Maximum Likelihood method and Tamura-Neimodel. Asterisk indicates the strains used in this study. “(T)” indicates the type strains. [file Image_4.JPEG]

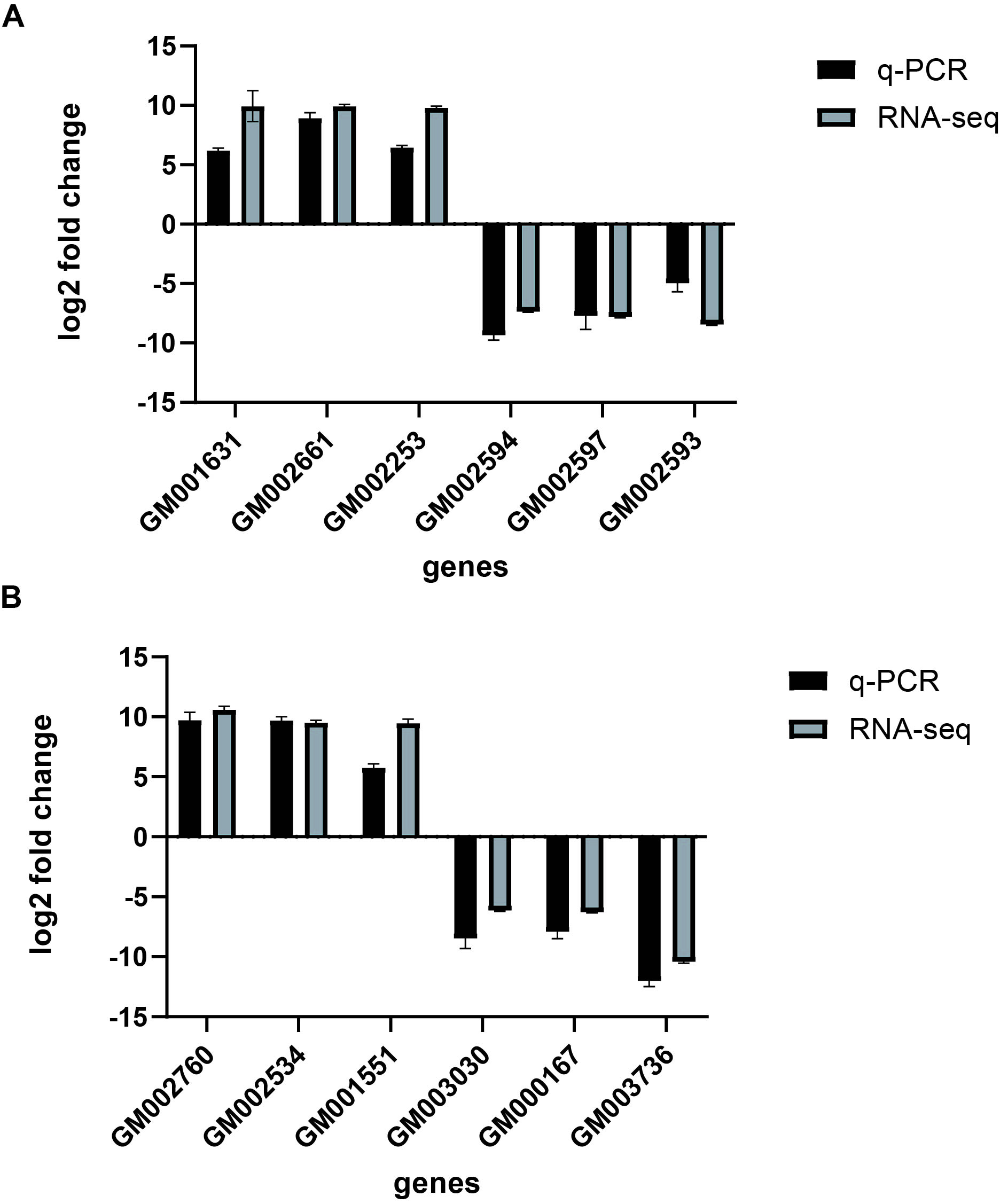

Supplement: SUPPLEMENTARY FIGURE S5 — qRT-PCR was employed to validate the RNA-Seq data. Six genes exhibited differential expression under the conditions in vivo and in vitro were selected respectively in SM and DQ. (A) Validation of gene expression levels in SM. (B) Validation of gene expression levels in DQ. [file Image_5.JPEG]

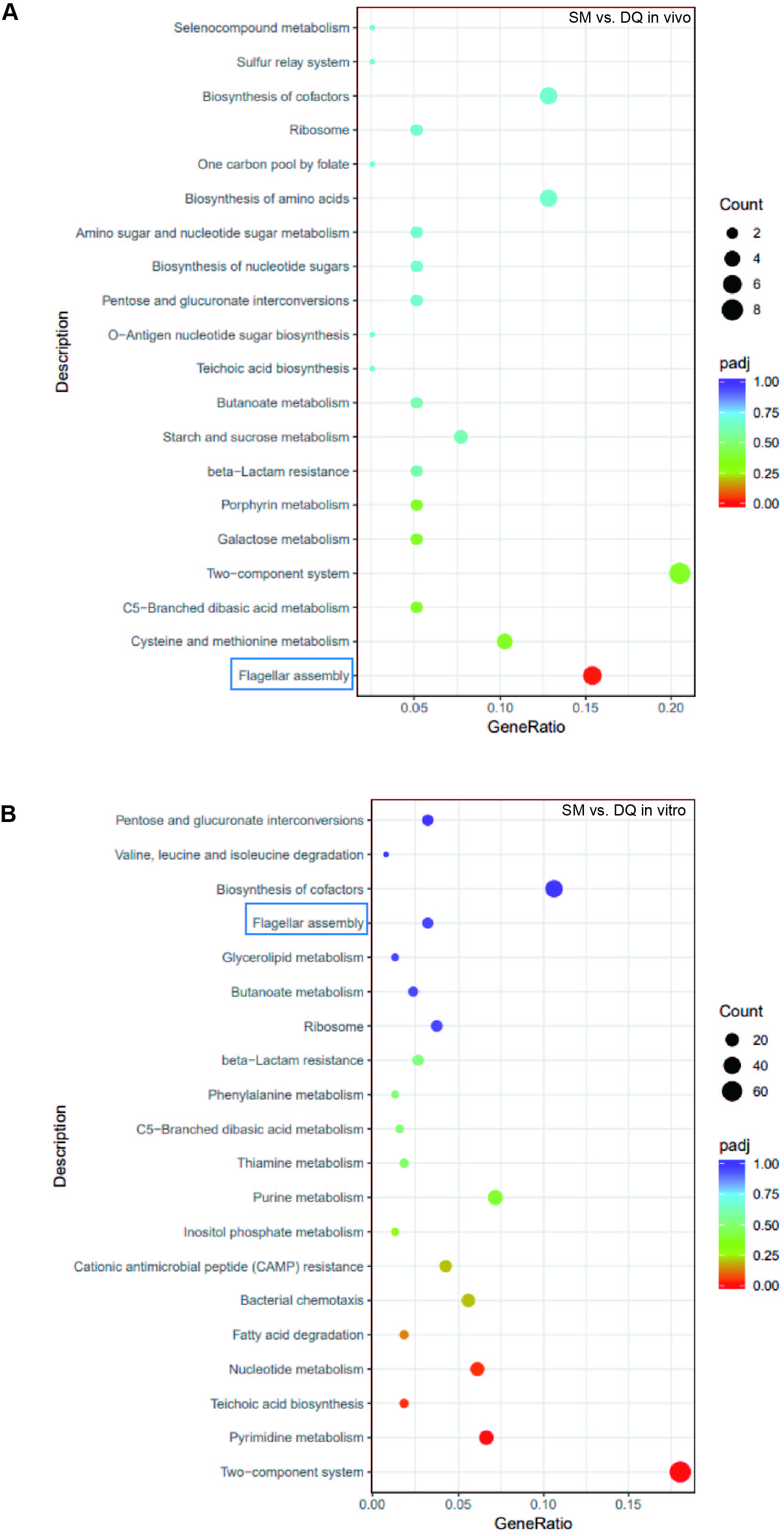

Supplement: SUPPLEMENTARY FIGURE S6 — KEGG enrichment analyses. (A) KEGG enrichment analysis of differentially expressed genes of SM compared to DQ recovered from potato stems after 24h infection. (B) KEGG enrichment analysis of differentially expressed genes of SM compared to DQ in NB medium. [file Image_6.JPEG]

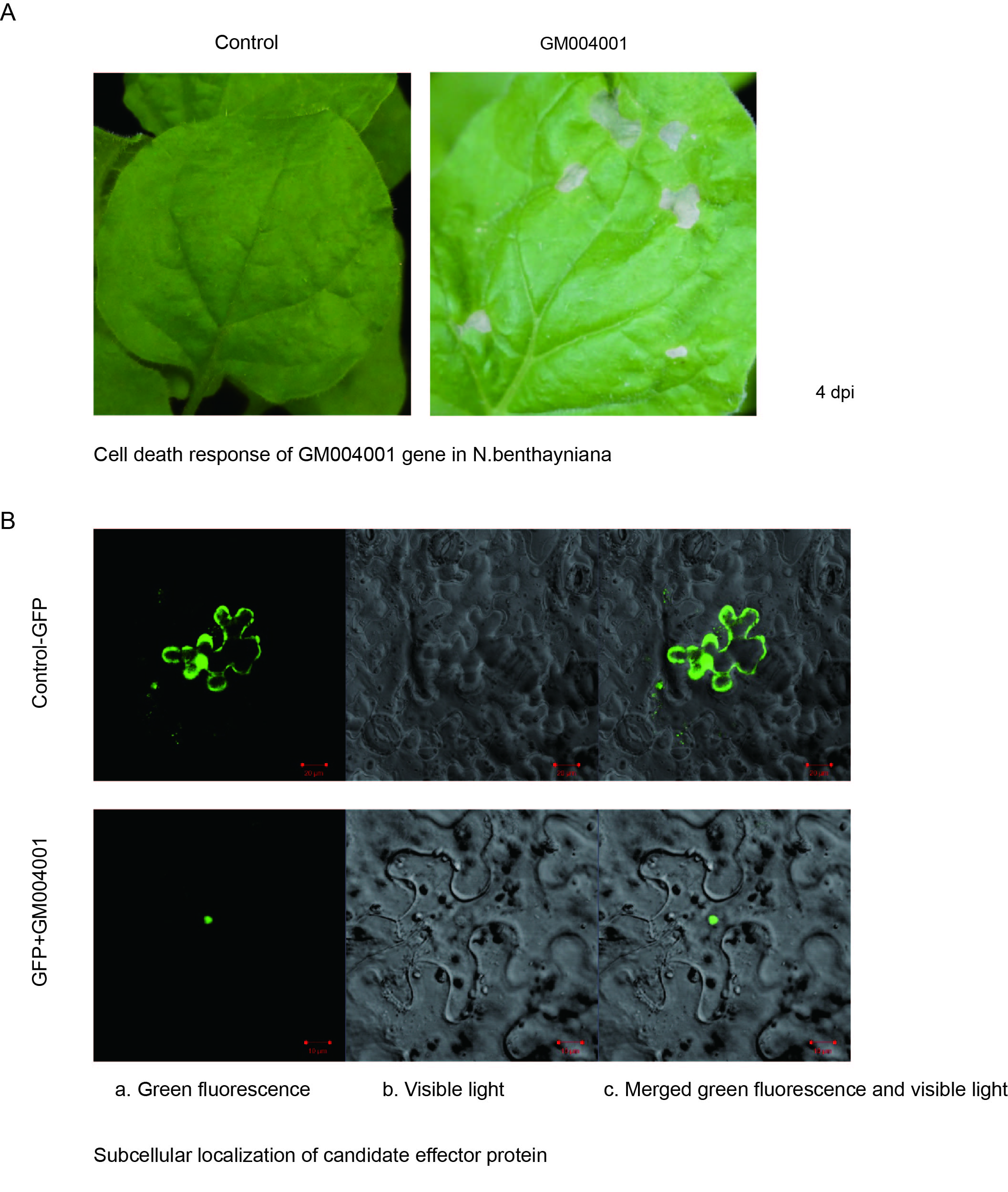

Supplement: SUPPLEMENTARY FIGURE S7 — Heterologously expressed GM4001 causes cell death and subcellular localization of GM4001. (A) Agrobacterium mediated transformation of tobacco leaves for expression of GM4001 gene. (B) Subcellular localization of GM4001. [file Image_7.JPEG]
