# Supplementary material for: Comparative genomic and transcriptome analyses of two Pectobacterium brasiliense strains revealed distinct virulence determinants and phenotypic features
Source: Front Microbiol. 2024 May 10;15:1362283. doi: 10.3389/fmicb.2024.1362283 (PMC11116658; doi:10.3389/fmicb.2024.1362283)
Supplement: Supplementary file 9 [file Table_1.DOCX]

| **Features** | **SM** | **DQ** | **SX309** | **1692** |
| --- | --- | --- | --- | --- |
| Size (bp) | 4953627 | 4764461 | 4966299 | 4851982 |
| G+C content (%) | 52.03 | 52.13 | 52.18 | 52 |
| Total gene number | 4390 | 4331 | 4455 | 4325 |
| Protein-coding genes | 4285 | 4227 | 4351 | 4219 |
| Transfer RNA | 77 | 76 | 76 | 77 |

**Table 1** Genomic features of *Pectobacterium brasiliense* SM, DQ, SX309 and 1692.
