# Supplementary material for: Comparative genomic and transcriptome analyses of two Pectobacterium brasiliense strains revealed distinct virulence determinants and phenotypic features
Source: Front Microbiol. 2024 May 10;15:1362283. doi: 10.3389/fmicb.2024.1362283 (PMC11116658; doi:10.3389/fmicb.2024.1362283)
Supplement: Supplementary file 10 [file Table_2.DOCX]

**Table S1** Statistical analysis of genome features

| **Attribute** | **Value**  **SM DQ** | **% of total**  **SM DQ** |
| --- | --- | --- |
| DNA scaffolds | 1 1 | 100.00 100.00 |
| RNA genes | 127 123 | 2.85 2.78 |
| Genes with Pfam domains | 3018 2984 | 67.77 67.51 |
| Genes with signal peptides | 305 282 | 6.85 6.38 |
| Genes assigned to KEGGs | 4254 4142 | 95.53 93.71 |
| CRISPR repeats | 3 3 | - - |
