# Supplementary material for: Comparative genomic and transcriptome analyses of two Pectobacterium brasiliense strains revealed distinct virulence determinants and phenotypic features
Source: Front Microbiol. 2024 May 10;15:1362283. doi: 10.3389/fmicb.2024.1362283 (PMC11116658; doi:10.3389/fmicb.2024.1362283)
Supplement: Supplementary file 11 [file Table_3.DOCX]

**Table S2** Percentage of average nucleotide identities (ANI)^a^ and in silico DNA-DNA hybridization (DDH)^b^ among SM, DQ and 1692 genomes

| Query genome  Reference genome | SM | DQ | 1692 (type strain) |
| --- | --- | --- | --- |
| SM |  | 97.27  86* | 96.42  79.2* |
| DQ | 97.27  86* |  | 96.09  80.5* |
| 1692 (type strain) | 96.42  79.2* | 96.09  80.5* |  |

^a^ANI values were computed for pairwise genome comparison with using JSpeciesWS. The percentage of ANI was shown on the top.

^b^In silico DNA-DNA hybridization was calculated by using Genome-to-Genome Distance Calculator (GGDC). The percentage of DDH was shown on the bottom with asterisk.
