# Supplementary material for: Comparative genomic and transcriptome analyses of two Pectobacterium brasiliense strains revealed distinct virulence determinants and phenotypic features
Source: Front Microbiol. 2024 May 10;15:1362283. doi: 10.3389/fmicb.2024.1362283 (PMC11116658; doi:10.3389/fmicb.2024.1362283)
Supplement: Supplementary file 12 [file Table_4.DOCX]

**Table S3** Numbers of specific genes of SM and DQ strains associated with KEGG function

| Category | Number of genes | | Description |
| --- | --- | --- | --- |
|  | SM | DQ |  |
| Cellular Processes | 1 | 1 | Cellular community - prokaryotes |
|  | 1 | 0 | Cell motility |
| Environmental Information Processing | 3 | 10 | Membrane transport |
|  | 1 | 0 | Signal transduction |
| Human Diseases | 0 | 3 | Infectious diseases: Bacterial |
|  | 5 | 1 | Drug resistance |
| Metabolism | 9 | 1 | Carbohydrate metabolism |
|  | 1 | 0 | Energy metabolism |
|  | 0 | 4 | Biosynthesis of other secondary metabolites |
|  | 1 | 1 | Metabolism of cofactors and vitamins |
|  | 1 | 0 | Nucleotide metabolism |
|  | 0 | 1 | Metabolism of other amino acids |
|  | 1 | 4 | Glycan biosynthesis and metabolism |
|  | 1 | 2 | Lipid metabolism |
| NA | 454 | 353 |  |
