# Supplementary material for: Comparative genomic and transcriptome analyses of two Pectobacterium brasiliense strains revealed distinct virulence determinants and phenotypic features
Source: Front Microbiol. 2024 May 10;15:1362283. doi: 10.3389/fmicb.2024.1362283 (PMC11116658; doi:10.3389/fmicb.2024.1362283)
Supplement: Supplementary file 13 [file Table_5.DOCX]

**Table S5** Identification of secretion system genes in SM and DQ

| **Secretion systems** | **SM** | | **DQ** | |
| --- | --- | --- | --- | --- |
|  | **Gene ID** | **Number** | **Gene ID** | **Number** |
| **Type I secretion system** |  | 4 |  | 3 |
| *lapB* | SM_GM001138 |  | NA |  |
| *tolC* | SM_GM000326 |  | DQ_GM000323 |  |
| *prtD* | SM_GM001548 |  | DQ_GM001461 |  |
| *prtD* | SM_GM001703 |  | DQ_GM001621 |  |
| **Type II secretion system** |  | 17 |  | 16 |
| *pulF* | SM_GM000541 |  | NA |  |
| *tadB* | SM_GM000806 |  | DQ_GM000699 |  |
| *tadC* | SM_GM000807 |  | DQ_GM000700 |  |
| *gspN* | SM_GM003098 |  | DQ_GM002969 |  |
| *gspM* | SM_GM003099 |  | DQ_GM002970 |  |
| *gspL* | SM_GM003100 |  | DQ_GM002971 |  |
| *gspK* | SM_GM003101 |  | DQ_GM002972 |  |
| *gspJ* | SM_GM003102 |  | DQ_GM002973 |  |
| *gspI* | SM_GM003103 |  | DQ_GM002974 |  |
| *gspH* | SM_GM003104 |  | DQ_GM002975 |  |
| *gspG* | SM_GM003105 |  | DQ_GM002976 |  |
| *gspF* | SM_GM003106 |  | DQ_GM002977 |  |
| *gspE* | SM_GM003107 |  | DQ_GM002978 |  |
| *gspE* | SM_GM003720 |  | DQ_GM003620 |  |
| *gspD* | SM_GM003108 |  | DQ_GM002979 |  |
| *gspC* | SM_GM003109 |  | DQ_GM002980 |  |
| *gspB* | SM_GM003112 |  | DQ_GM002983 |  |
| **Type III secretion system** |  | 14 |  | 14 |
| *dspF* | SM_GM002358 |  | DQ_GM002253 |  |
| *hrcC* | SM_GM002370 |  | DQ_GM002269 |  |
| *hrpE* | SM_GM002373 |  | DQ_GM002272 |  |
| *hrpB* | SM_GM002376 |  | DQ_GM002275 |  |
| *hrpL* | SM_GM002382 |  | DQ_GM002282 |  |
| *hrpJ* | SM_GM002384 |  | DQ_GM002283 |  |
| *hrcV* | SM_GM002385 |  | DQ_GM002284 |  |
| *hrcN* | SM_GM002387 |  | DQ_GM002286 |  |
| *sctQ* | SM_GM002390 |  | DQ_GM002289 |  |
| *hrcR* | SM_GM002391 |  | DQ_GM002290 |  |
| *hrcS* | SM_GM002392 |  | DQ_GM002291 |  |
| *hrcT* | SM_GM002393 |  | DQ_GM002292 |  |
| *hrcU* | SM_GM002394 |  | DQ_GM002293 |  |
| *hrpQ* | SM_GM002386 |  | DQ_GM002285 |  |
| **Type VI secretion system** |  | 31 |  | 29 |
| *vgrG* | SM_GM000065 |  | DQ_GM000067 |  |
| *vgrG* | NA |  | DQ_GM000093 |  |
| *vgrG* | SM_GM002366 |  | DQ_GM002265 |  |
| *vgrG* | SM_GM002660 |  | DQ_GM002533 |  |
| *vgrG* | SM_GM003406 |  | DQ_GM003343 |  |
| *hcp* | SM_GM000066 |  | DQ_GM000068 |  |
| *hcp* | SM_GM000123 |  | NA |  |
| *hcp* | SM_GM000466 |  | DQ_GM000454 |  |
| *hcp* | NA |  | DQ_GM000678 |  |
| *hcp* | SM_GM001109 |  | NA |  |
| *hcp* | SM_GM001112 |  | DQ_GM001015 |  |
| *hcp* | SM_GM001764 |  | DQ_GM001574 |  |
| *hcp* | SM_GM001774 |  | NA |  |
| *hcp* | SM_GM002661 |  | DQ_GM002534 |  |
| *hcp* | SM_GM003407 |  | DQ_GM003293 |  |
| *hcp* | SM_GM003427 |  | DQ_GM003311 |  |
| *hcp* | SM_GM003644 |  | DQ_GM003547 |  |
| *hcp* | SM_GM004231 |  | DQ_GM004201 |  |
| *hcp* | SM_GM004273 |  | NA |  |
| *impA* | SM_GM003409 |  | DQ_GM003295 |  |
| *tssM* | SM_GM003410 |  | DQ_GM003296 |  |
| *tssA* | SM_GM003411 |  | DQ_GM003297 |  |
| *vasI* | SM_GM003412 |  | DQ_GM003298 |  |
| *tssH* | SM_GM003414 |  | DQ_GM003300 |  |
| *impK* | SM_GM003415 |  | DQ_GM003301 |  |
| *tssK* | SM_GM003416 |  | DQ_GM003302 |  |
| *tssJ* | SM_GM003417 |  | DQ_GM003303 |  |
| *impI* | SM_GM003418 |  | DQ_GM003304 |  |
| *tssG* | SM_GM003419 |  | DQ_GM003305 |  |
| *tssF* | SM_GM003420 |  | DQ_GM003306 |  |
| *tssE* | SM_GM003421 |  | DQ_GM003307 |  |
| *impC* | SM_GM003422 |  | DQ_GM003308 |  |
| *impB* | SM_GM003423 |  | DQ_GM003309 |  |
