# Supplementary material for: Comparative genomic and transcriptome analyses of two Pectobacterium brasiliense strains revealed distinct virulence determinants and phenotypic features
Source: Front Microbiol. 2024 May 10;15:1362283. doi: 10.3389/fmicb.2024.1362283 (PMC11116658; doi:10.3389/fmicb.2024.1362283)
Supplement: Supplementary file 15 [file Table_7.DOCX]

**Table S7 Strains and plasmids used in this study.**

| Name | Description | Source |
| --- | --- | --- |
| ***E. coli* strain** | | |
| DH5alpha competent cell | general cloning strain | TransGen |
| [S17-1 λpir](http://www.maokangbio.com/productView.action?id=11415) competent cell | general cloning strain | Weidibio |
| RHO competent cell | general cloning strain | Lab collection |
| ***Pectobacterium* strains** | | |
| *Pectobacterium brasiliense* SM | The strain causes potato soft rot disease | This study |
| *Pectobacterium brasiliense* DQ | The strain causes potato soft rot disease | This study |
| GH12OE | GH12 overexpression DQ strain | This study |
| ΔGH12 | GH12 knock-out SM strain | This study |
| CΔGH12 | GH12-complemented expression SM strain | This study |
| ***Agrobacterium* strain** | | |
| GV3101 | Transfection | Lab collection |
| **Plasmids** | | |
| pH-2-GH12 | The expression of GH12 is driven by the promoter itself. | This study |
| pCAMBIAsuper1300-GFP | Plant expression vector | Lab collection |
| pLP12 | Suicide plasmid | miaolingbio |
