# Supplementary material for: Comparative genomic and transcriptome analyses of two Pectobacterium brasiliense strains revealed distinct virulence determinants and phenotypic features
Source: Front Microbiol. 2024 May 10;15:1362283. doi: 10.3389/fmicb.2024.1362283 (PMC11116658; doi:10.3389/fmicb.2024.1362283)
Supplement: Supplementary file 16 [file Table_8.DOCX]

**Table S8 Primers used in this study.**

| **Primers for construction of overexpression plasmid** | |
| --- | --- |
| GH12-F | GCATATGCATGTCGGCAGAGGATCCAATAACAATAAACTGTTAAGCACAC |
| GH12-R | AAAGCTTCTGCAGGAGCTCGAATTCGGGAAATTCCTGATTAGCTGAG |
| CDS sequence of GH12 | ATGCTTATTATGAATACTCATTCGCCGCGTACTTTCCGCACCTTTCTCCCCGCAATTCTGTCTGTTTTATTACTCTCGCCCTTAACGGTTTCTGCCGCCAGTTCCTCAAAGGACGCAGATAAACTCTATTTTGAAAATAATAAATATTATGTATTCAATAACGTCTGGGGAAAAGATGAAGTAAAAGGGTGGCAGCAAACCGTTTTCTATAATAGCCCAACCAGCATGGGGTGGAACTGGCACTGGCCAAGCAGTAGCCACAGCGTTAAAGCTTATCCATCACTGGTGAGCGGCTGGCACTGGACGGCAGGTTATACGGAAAATAGTGGATTGCCGATAAAATTATCCAGTAATAAAAGCATTACCAGCAACGTCACTTATTCCATCAAATCTACCGGCACGTATAATGCTACTTATGACATATGGTTCCACACGACCGATAAAGCCAGTTGGGACACGGCTCCTACCGATGAATTAATGATCTGGCTAAATAATACCAATGCAGGCCCAGCCGGTGATTATATTGAAACAGTTTTCCTTGGAGGCAGTAACTGGAATGTATTCAAAGGTTGGATCAATGCGGGTAATGGCAAAGGGTGG  AACGTATTTTCCTTTGTCCGCACCTCTAATACCAACAACGCATCACTCAATATTCGCCATTTCACCAACTATCTAGTGGGAACCAAGAAATGGATGAGTAATACAAAATATATCAGCAGCGTTCAGTTCGGTACCGAGATCTTTGGCGGTGATGGACAGATTGACATCACCAAGTGGAGCGTAGACGTAAAATAA |
| **Primers for construction of knock-out plasmid** | |
| ΔGH12-LBF | GCAGGAATCTAGACCTTGAGTCGAATTCGACCAGCGCCACATTTGGCATC |
| ΔGH12-LBR | GATATAGACGCGCTCCTTTTAATATATGCTCGGGATGTTTCACTC |
| ΔGH12-RBF | ATTAAAAGGAGCGCGTCTATATCCTTCTGGTCC |
| ΔGH12-RBR | TTAACCCTCACTAAAGGGAACAGCTAGCCATAACCAGCCTGGTAAGCCGC |
| **Primers used for qRT-PCR verification** | |
| Primers for verification of SM | |
| GM001631F | AACTGGATGGAAGCAACAGCGTATT |
| GM001631R | GACAGCATAGATAGCAAGCCGACTC |
| GM002661F | CTGTCGGCAACATCTATGTGGAAGG |
| GM002661R | TCGGTCAGCACGGTAGAGAAGAAG |
| GM002253F | GGACGACATCTGTTACGGCTACG |
| GM002253R | CACGCCACGGTAATCCAGTTGAT |
| GM002594F | GGTTCACAGTCGGTCGTCTCAC |
| GM002594R | GACAAGGTCATGCCATCCAGGTT |
| GM002597F | GTATGGAGTTCGGCTTCGGTGAG |
| GM002597R | TCGCTCTCGGAATAGATGTCCTTGA |
| GM002593F | GCGAGTCTGGAGTCCAAGCTAATC |
| GM002593R | GGCTCTTCTTCTACCGAGGATTGC |
| Primers for verification of DQ | |
| GM002760F | CCTCTGTATTTATGCTTGCCAACGAAAC |
| GM002760R | CTGACTGAAGTTGTGATGCTGGTTCT |
| GM002534F | CTGTCGGCAACATCTATGTGGAAGG |
| GM002534R | ATCGGTCAGCACGGTAGAGAAGAA |
| GM001551F | AACTGGATGGAAGCAACAGCGTATT |
| GM001551R | GACAGCATAGATAGCAAGCCGACTC |
| GM003030F | AACGGTCAGTATGAGGTCAACTTCC |
| GM003030R | TGATGAACAGCAGGCGATGTAGC |
| GM000167F | CGCTGGTCTACGGTCTGTATCAC |
| GM000167R | AATGTGTGGGTTCGGATAGGTTGAG |
| GM003736F | TGCGTTGCTTAGCCAGTCTTCA |
| GM003736R | ATGCGGATTCAGCCAGGTCAC |
| House-keeping genes | |
| *gapA*-F | TCGGTATCGTTGAAGCACTGATGAC |
| *gapA*-R | TTGCCGTTCAGCTCAGGAATCAC |
| **Primers used for subcellular localization** | |
| 4001F | ATACACCAAATCGACTCTAGAATGATGGAAAATACGCCGTCTG |
| 4001R | GCCCTTGCTCACCATGGTACCTGCAGAACGTTGCTGCATC |
